# A Microtubule-Associated Protein Is Essential for Malaria Parasite Transmission

**DOI:** 10.1128/mbio.03318-22

**Published:** 2023-01-10

**Authors:** Jan Stephan Wichers-Misterek, Annika M. Binder, Paolo Mesén-Ramírez, Lilian Patrick Dorner, Soraya Safavi, Gwendolin Fuchs, Tobias L. Lenz, Anna Bachmann, Danny Wilson, Friedrich Frischknecht, Tim-Wolf Gilberger

**Affiliations:** a Centre for Structural Systems Biology, Hamburg, Germany; b Bernhard Nocht Institute for Tropical Medicine, Hamburg, Germany; c Biology Department, University of Hamburg, Hamburg, Germany; d Integrative Parasitology, Department of Infectious Diseases, Heidelberg University Medical School, Heidelberg, Germany; e Research Unit for Evolutionary Immunogenomics, Department of Biology, University of Hamburg, Hamburg, Germany; f German Center for Infection Research, Partner Site Hamburg-Borstel-Lübeck-Riems, Hamburg, Germany; g Research Centre for Infectious Diseases, School of Biological Sciences, University of Adelaide, Adelaide, South Australia, Australia; h Burnet Institute, Melbourne, Victoria, Australia; i Institute for Photonics and Advanced Sensing, University of Adelaide, Adelaide, South Australia, Australia; j German Center for Infection Research, Partner Site Heidelberg, Heidelberg, Germany; NIAID/NIH

**Keywords:** gametocytogenesis, malaria, microtubule, *Plasmodium falciparum*

## Abstract

Mature gametocytes of Plasmodium falciparum display a banana (falciform) shape conferred by a complex array of subpellicular microtubules (SPMT) associated with the inner membrane complex (IMC). Microtubule-associated proteins (MAPs) define MT populations and modulate interaction with pellicular components. Several MAPs have been identified in Toxoplasma gondii, and homologues can be found in the genomes of *Plasmodium* species, but the function of these proteins for asexual and sexual development of malaria parasites is still unknown. Here, we identified a novel subpellicular MAP, termed SPM3, that is conserved within the genus *Plasmodium*, especially within the subgenus *Laverania*, but absent in other Apicomplexa. Conditional knockdown and targeted gene disruption of *Pfspm3* in Plasmodium falciparum cause severe morphological defects during gametocytogenesis, leading to round, nonfalciform gametocytes with an aberrant SPMT pattern. In contrast, *Pbspm3* knockout in Plasmodium berghei, a species with round gametocytes, caused no defect in gametocytogenesis, but sporozoites displayed an aberrant motility and a dramatic defect in invasion of salivary glands, leading to a decreased efficiency in transmission. Electron microscopy revealed a dissociation of the SPMT from the IMC in *Pbspm3* knockout parasites, suggesting a function of SPM3 in anchoring MTs to the IMC. Overall, our results highlight SPM3 as a pellicular component with essential functions for malaria parasite transmission.

## INTRODUCTION

Despite significant progress in combating malaria, this disease remains a huge burden on health systems in tropical countries worldwide, with an estimated 241 million cases and 627,000 deaths in 2020 (1). The deadliest species, Plasmodium falciparum, accounts for almost 99% of malaria-associated fatalities. P. falciparum emerged from the *Laverania* clade, a group of ape-infecting *Plasmodium* parasites comprising at least seven cryptic species infecting chimpanzees, gorillas, and bonobos (2–5). This clade represents a subgroup among hundreds of known *Plasmodium* species infecting various vertebrate hosts, including birds, reptiles, rodents, and humans. Malaria parasites evolved and developed a complex life cycle alternating between invertebrate vectors and vertebrate hosts, leading to the evolution of several highly specialized and morphologically distinct developmental stages that are adapted to a specific niche within different host tissue (6). The drastic morphological changes across the life cycle and the morphogenesis of the different stages are driven by reorganization of cytoskeletal structures (7). A key structural feature driving the transition between different life cycle stages is the unique three-membrane pellicle of these cells, which consists of the parasite plasma membrane (PPM) and a double-membrane structure, specific to alveolates, termed the inner membrane complex (IMC), or alveoli, which are intramembranous vesicles underlying the PPM and are likely linked to the subpellicular microtubule (SPMT) cytoskeleton (8–11). The different arrangements of the pellicle and its components, especially the IMC and the SPMTs, in structurally distinct malaria parasite stages may have evolved to fulfill the unique requirements for invasion and survival in different host cells (reviewed in reference 12). In invasive stages, SPMTs originate at the apical polar ring, an apicomplexan-specific microtubule organizing center (MTOC), and extend to the parasite’s posterior end in close association with the cytosolic face of the parasite pellicle (11).

The number of SPMTs varies across life cycle stages. For instance, merozoites of P. falciparum possess 2 to 4 SPMTs (13, 14), whereas ookinetes have 60, sporozoites 14, and gametocytes 21 SPMTs (11). SPMTs of P. falciparum are extremely stable, as exemplified by their resistance to classic microtubule-depolymerizing agents (15). Recent studies showed polyglutamylation of SPMTs in merozoites (16) and gametocytes (17) and suggested its involvement in SPMT stability (16). A second population of microtubules, the spindle microtubules, is necessary to coordinate chromosome segregation, and these extend from a single MTOC throughout the nucleus (13, 18). Gametocytes, the blood-circulating sexual stages critical for malaria transmission, also possess an elaborate IMC and an array of SPMTs (19–22). Strikingly, mature gametocytes of P. falciparum and the other species of the clade *Laverania* display a banana (falciform) shape, while round ameboid gametocytes are observed for many rodent-infecting species, such as Plasmodium berghei, and in other human-infecting species, like Plasmodium vivax (22–25). The underlying molecular differences between species that define this morphology are not clearly understood.

The elongated cell shape of P. falciparum gametocytes is conferred by the assembly of an array of SPMTs recruited to the nascent IMC in early gametocyte stages, which extend laterally as gametocytes mature (20). Here, the SPMTs are 27 to 38 nm in diameter (26) and linearly spaced 10 to 25 nm apart (22, 24). In contrast to invasive stages, such as sporozoites, merozoites, and ookinetes, gametocytes lack apical polarity and an apical polar ring from which SPMTs emanate. A recent study reports the nucleation of SPMTs in gametocytes at the outer centriolar plaque, a nonmitotic MTOC embedded in the nuclear membrane of the parasite (17). The SPMT network disassembles in stage V gametocytes, providing an increased deformability that is expected to permit transmigration across the endothelial barrier and exit from the bone marrow (27, 28). Although robust gametocyte motility *in vivo* has not been reported, their elongated morphology, which is similar to motile “zoite” forms, may contribute to movement between tissues and vessels (29).

Numerous studies in the model apicomplexan organism Toxoplasma gondii, identified several microtubule-associated proteins (MAPs) decorating the SPMTs, including *Tg*SPM1/2 (30), *Tg*TrxL1/2, and *Tg*TLAP1/2/3/4 (31, 32). MAPs are expected to define SPMT subpopulations with different properties and to modulate microtubule stability, mechanical properties, and resistance to depolymerization (30, 32–35). Some of these MAPs, such as *Tg*TrxL1, *Tg*TrxL2, and *Tg*SPM1, are known to localize within the lumen of SPMTs (36), while others might interact with their external surface. Amino acid repeats in the *Tg*MAPs are important for microtubule localization and tubulin binding (30). *Tg*SPM1 and *Tg*TrxL1 are important for stability of the SPMTs but are not essential for parasite development, suggesting remarkable functional redundancy (30, 36). *Tg*AC9 and *Tg*AC10 were shown to be required for the organization of the SPMTs at the apical cap and mediate interaction to IMC components (37), while *Tg*DCX has been shown to modify the structure of tubulin in the conoid (38).

Homologues of most of the *Tg*MAPs can be found in the genomes of *Plasmodium* species, but none has been experimentally characterized for either asexual or sexual developmental stages so far. The presence of *Plasmodium* MAPs within the SPMT lumen was suggested previously (39, 40), but SPMT structures resembling those expected for P. falciparum SPM1 (*Pf*SPM1) and *Pf*TRXL1 in the SPMT lumen only recently identified by cryo-electron microscopy (cryo-EM) and modeling, supporting the idea that these proteins have a role in SPMT function (26).

Although the findings of Ferreira et al. (26) and the presence of several homologues to T. gondii MAPs in the P. falciparum genome suggest that potentially many *Pf*MAPs might have functions similar to those in T. gondii, differences in the malaria parasite life cycle and the structure of microtubules open up the possibility that they have evolved MAPs with unique functions.

Here, we identified SPM3 (PF3D7_1327300/PBANKA_1342500) as a novel MAP that is highly conserved within *Plasmodium* spp., especially within the clade *Laverania*, but absent in other Apicomplexa such as T. gondii. Characterization of SPM3 reveals its importance in falciform gametocyte morphology and sexual development in P. falciparum, as well as its role in the transmission of P. berghei sporozoites.

## RESULTS

### *Pf*SPM3 is a *Plasmodium*-specific MAP in P. falciparum merozoites.

**FIG 1:**
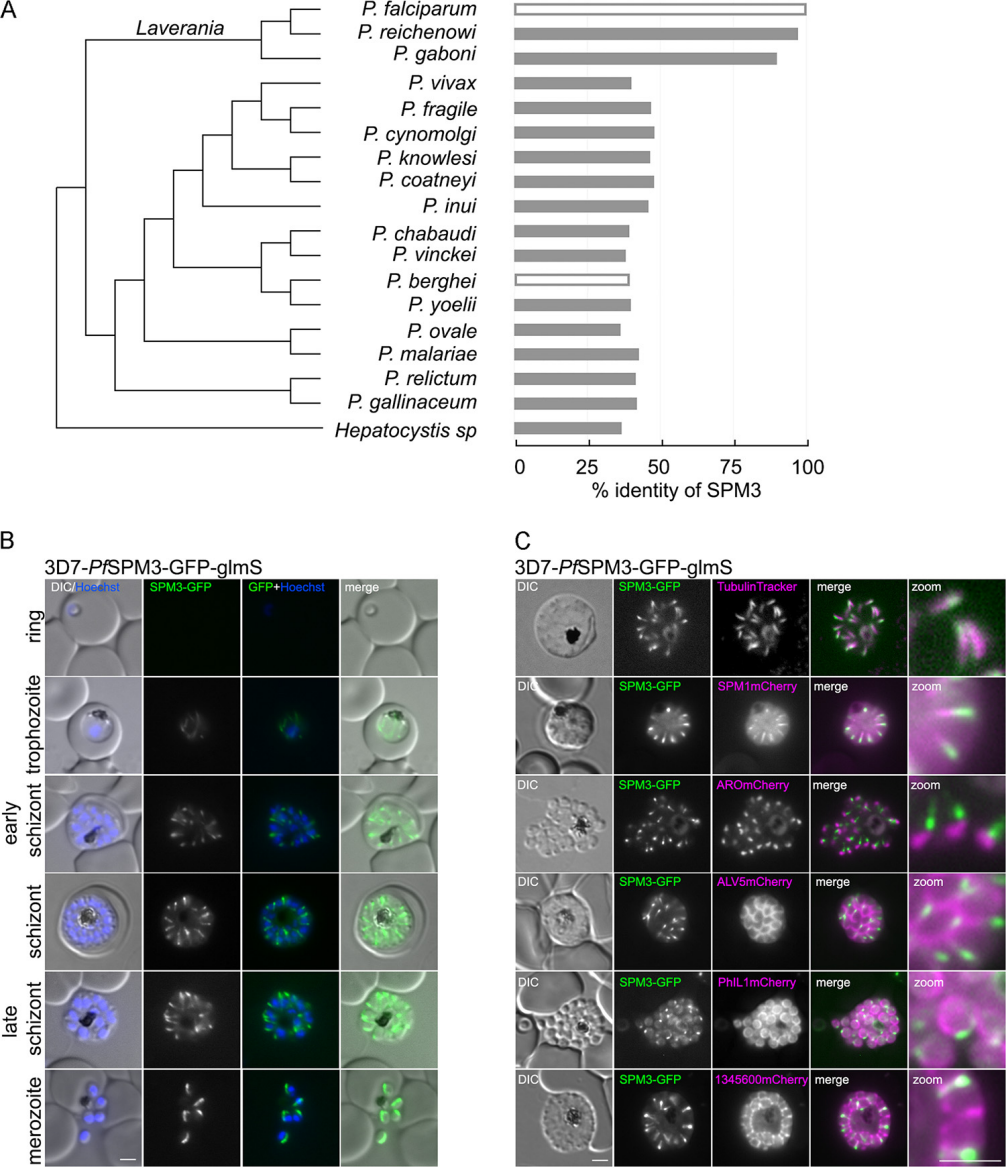
*Pf*SPM3 localizes to the SPMTs of P. falciparum merozoites. (A) Phylogenetic relatedness of sequences homologous to SPM3 across species (fast minimum evolution tree based on Grishin protein distance) and similarity along homologous sequence stretches. (B) Localization of *Pf*SPM3-GFP by live-cell microscopy during intracellular development cycle in 3D7-*Pf*SPM3-GFP-glmS. (C) Colocalization of *Pf*SPM3-GFP with TubulinTracker, microtubule-associated protein *Pf*SPM1mCherry, the rhoptry marker protein ARO-mCherry, and the IMC marker proteins ALV5mCherry, PhIL1mCherry, and PF3D7_ 1345600mCherry. Nuclei were stained with Hoechst 33342. Bars, 2 μm. Zoom factor, 400%, with a scale bar of 1 μm. DIC, differential inference contrast.

PF3D7_1327300 was initially identified as a putative interaction partner of the IMC protein *Pf*PhIL1 (41). Phylogenetic analysis revealed orthologs of *Pf*SPM3 with high similarity (>89% amino acid similarity) in Plasmodium reichenowi and P. gaboni (Fig. 1A). Other *Plasmodium* species also appear to have homologous proteins, but with significantly lower similarity (<50% amino acid similarity) than in *Laverania* parasites. The only homologous protein sequence outside the genus *Plasmodium* was found in *Hepatocystis* sp. (Fig. 1A).

To test putative IMC association and to investigate the physiological role of this protein in P. falciparum, we first generated transgenic parasites expressing a C-terminal green fluorescent protein (GFP) tag. Genomic integration of an SLI (selection-linked integration)-based plasmid (42) into the *spm3* locus of the 3D7 parental strain was verified by PCR (see Fig. S1A in the supplemental material). Expression and localization of the fusion protein were analyzed by fluorescence microscopy. The GFP fusion protein was detectable from the trophozoite stage onward, in agreement with its transcriptional profile (43, 44). Unexpectedly, its localization did not resemble the localization of any IMC-resident proteins but was reminiscent of SPMT distribution (45) (Fig. 1B). Localization of GFP-tagged *Pf*SPM3 at the microtubules was visualized by colocalization with TubulinTracker (Fig. 1C, first row), showing partial overlap with a more pronounced signal of *Pf*SPM3 toward one end of the SPMT. This SPMT association was substantiated by colocalization with an overexpressed mCherry-tagged *Pf*SPM1 (PF3D7_0909500) (Fig. 1C, second row), the homologue of *Tg*SPM1 (30), which showed a distribution similar to that of TubulinTracker, with some cytosolic background.

10.1128/mbio.03318-22.1 FIG S1 Validation of generated transgenic cell lines. (A) Confirmatory PCR of unmodified WT and transgenic knock-in (KI) cell lines to check for genomic integration at the 3′ and 5′ ends of the locus. Positions of the primers used are indicated with numbered arrows. (B) Schematic representation of KI and TGD strategy using the SLI system (42). Pink, human dihydrofolate dehydrogenase (hDHFR); grey, homology region (HR); green, GFP tag; dark grey, T2A skip peptide; blue, neomycin resistance cassette; orange, *glmS* sequence. Stars indicate stop codons, and arrows depict primers (P1 to P4) used for the integration check PCR. Download FIG S1, TIF file, 0.7 MB. Copyright © 2023 Wichers-Misterek et al. 2023 Wichers-Misterek et al. https://creativecommons.org/licenses/by/4.0/ This content is distributed under the terms of the Creative Commons Attribution 4.0 International license.

Colocalization of *Pf*SPM3-GFP with *Pf*SPM1-mCherry or TubulinTracker underlined the more apical distribution of *Pf*SPM3 along SPMTs compared with *Pf*SPM1-mCherry and TubulinTracker. Based on these localization data, PF3D7_1327300 was named *Pf*SPM3 (for subpellicular microtubule protein 3), acknowledging the previously published SPMT-associated proteins *Tg*SPM1 and *Tg*SPM2 in T. gondii (30). To confirm the apical distribution of *Pf*SPM3-GFP on the SPMT, we colocalized the protein with the rhoptry bulb marker ARO-mCherry (46, 47) and found that the *Pf*SPM3-GFP signal is significantly concentrated toward the apical pole of the SPMT in nascent merozoites (Fig. 1C, third row). Finally, in order to investigate the previously described potential interaction of *Pf*SPM3 with *Pf*PhIL1 (41), *Pf*SPM3 was colocalized with three different IMC marker proteins: ALV5-mCherry (48, 49), PhIL1-mCherry (20, 50, 51), and PF3D7_1345600 mCherry (52) (Fig. 1B, fourth to sixth rows). Colocalization with these IMC markers shows, in agreement with the *Pf*SPM1 and TubulinTracker investigations, that *Pf*SPM3 lies in close proximity to, but spatially distinct from, the IMC.

### *Pf*SPM3 is dispensable for intraerythrocytic development.

**FIG 2:**
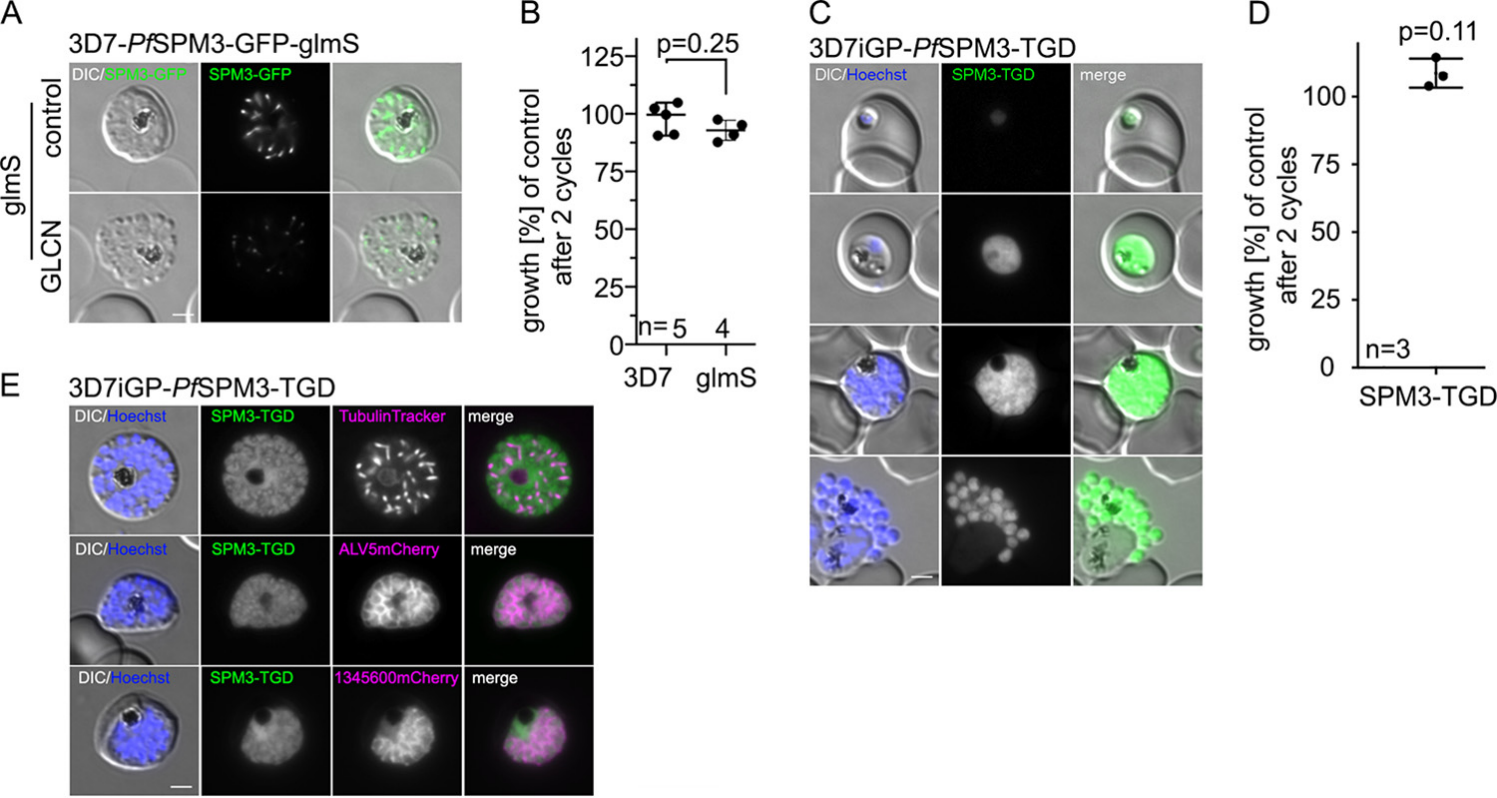
*Pf*SPM3 is dispensable for the asexual replication cycle. (A) Representative live-cell microscopy of 3D7-*Pf*SPM3-GFP-glmS schizonts cultured either with or without (control) 2.5 mM GLCN at 40 h after addition of GLCN. Scale bar, 2 μm. (B) Growth of 3D7 and 3D7-*Pf*SPM3-GFP-glmS parasites treated with or without 2.5 mM GLCN after two parasite replication cycles, as determined by flow cytometry. Shown are relative parasitemia values, which were obtained by dividing the parasitemia of GLCN-treated cultures by the parasitemia of the corresponding untreated ones. Values are means and standard deviations (SD) from independent growth experiments, with the number of experiments (n) indicated. *P* values were determined with a two-tailed unpaired *t* test with Welch’s correction. (C) Localization of truncated *Pf*SPM3-TGD-GFP fusion protein by live-cell microscopy across the intraerythrocytic development in the 3D7-iGP background. (D) Growth curves of 3D7-iGP-*Pf*SPM3-TGD versus 3D7-iGP parasites after two parasite replication cycles, as determined by flow cytometry. Three independent growth experiments were performed, and a summary is shown as percentage of growth compared to the parental parasite line. Values are means and SD from independent growth experiments, with the number of experiments (n) indicated. *P* values were determined with a one-sample *t* test. (E) Colocalization of the truncated *Pf*SPM3-TGD-GFP fusion protein with TubulinTracker and the IMC marker proteins ALV5mCherry and PF3D7_ 1345600mCherry. Nuclei were stained with Hoechst 33342. Bar, 2 μm. Zoom factor, 400%, with a scale bar of 1 μm.

To assess the function of *Pf*SPM3 in asexual development of P. falciparum parasites, a glucosamine-inducible degradation system was used to degrade *Pf*SPM3 mRNA (*glmS* ribozyme system) (53) (Fig. S1A and B). The *glmS* ribozyme was introduced upstream of the 3′ untranslated region in the modified *spm3* locus of the 3D7 parental strain. Although conditional knockdown of *Pf*SPM3-GFP upon addition of 2.5 mM glucosamine resulted in reduction of the GFP signal (Fig. 2A), it had no measurable effect on either parasite proliferation during asexual blood-stage parasite development (Fig. 2B) or schizont morphology (Fig. 2A). To exclude the possibility that residual amounts of *Pf*SPM3 upon knockdown might be sufficient for parasite development, we tested an additional approach using targeted gene disruption (TGD) (42) that truncates most of the protein and leads to the expression of only the N-terminal 136 amino acids instead of the 1,795-amino-acid wild-type (WT) *Pf*SPM3 protein (Fig. S1B). For further phenotypical characterization of this truncation, the cell line was generated in a 3D7-iGP background (54). Successful integration and consequent disruption of the *Pfspm3* gene were validated by PCR (Fig. S1A). Expression and localization of the truncated protein fragment were monitored by fluorescence microscopy. In contrast to full-length *Pf*SPM3, this truncated version is not connected with the SPMT but exhibits a cytosolic distribution (Fig. 2C). Nevertheless, and in agreement with the knockdown approach, these *Pf*SPM3-deficient parasites showed neither a growth nor a morphological phenotype (Fig. 2C and D). Congruently, these parasites revealed an SPMT and IMC architecture indistinguishable from that of wild-type parasites, as visualized by coexpression of the IMC marker proteins ALV5-mCherry (48, 49) and PF3D7_1345600-mCherry (52) or by costaining with TubulinTracker (Fig. 2E), confirming the dispensability of *Pf*SPM3 for asexual blood-stage development.

### *Pf*SPM3 is important for falciform gametocyte morphology.

**FIG 3:**
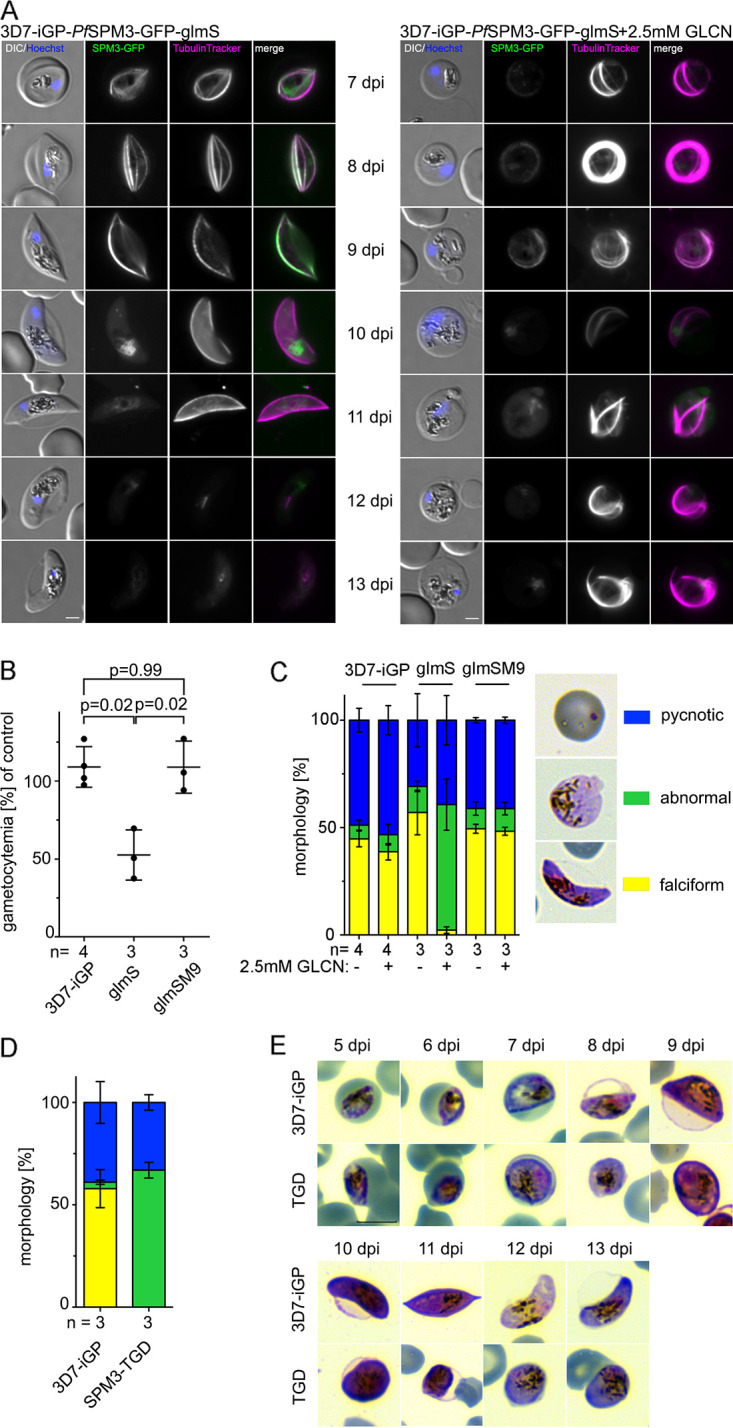
*Pf*SPM3 deficiency interferes with gametocytogenesis and leads to nonfalciform morphology. (A) Live-cell microscopy of 3D7-iGP-*Pf*SPM3-GFP gametocytes (day 7 to day 13 after gametocyte induction) cultured either with or without (control) 2.5 mM GLCN. Microtubules were visualized with TubulinTracker, and nuclei were stained with Hoechst 33342. Bar, 2 μm. (B) Gametocytemia at day 10 after gametocyte induction was determined by counting 1,096 to 2,632 (mean, 1,793) cells per condition in Giemsa-stained thin blood smears. The relative gametocytemia values were obtained by dividing the gametocytemia of GLCN-treated cultures by the gametocytemia of the corresponding untreated cultures. Values are means and SD from independent growth experiments, with the number of experiments (n) indicated. A two-tailed unpaired *t* test with Welch’s and Benjamini-Hochberg corrections was used to calculate multiplicity-adjusted *P* values for 3D7-iGP-*Pf*SPM3-GFP-glmS or 3D7-iGP-*Pf*SPM3-GFP-glmSM9 versus 3D7-iGP parasites all cultured with 2.5 mM GLCN. (C) Classification and quantification of gametocyte morphology within parasite populations of 3D7-iGP, 3D7-iGP-*Pf*SPM3-GFP-glmS, and 3D7-iGP-*Pf*SPM3-GFP-glmSM9 cultured either with or without (control) 2.5 mM GLCN. Between 589 and 2,632 erythrocytes and 19 to 69 (mean, 48) parasites per condition in Giemsa-stained thin blood smears were analyzed. Yellow, falciform; blue, pycnotic; green, abnormal. Representative images are shown on the right at day 10 after gametocyte induction. (D) Quantification of gametocyte morphology (yellow, falciform; blue, pycnotic; green, abnormal) at day 10 after gametocyte induction for 3D7-iGP (control) and 3D7-iGP-SPM3-TGD parasites. For each condition, the proportion of parasite stages in erythrocytes was determined in three independent experiments (total numbers of erythrocytes screened for 3D7-iGP were 2,694, 1,431, and 2,742; numbers for 3D7-iGP-SPM3-TGD were 1,328, 1,144, and 925) and displayed as a percentage. (E) Representative Giemsa smears of 3D7-iGP and 3D7-iGP-*Pf*SPM3-TGD stage II to V gametocytes (day 5 to day 13 after gametocyte induction). Bar, 5 μm.

In addition to the 3D7-based transgenic parasites, we generated a corresponding cell line in the 3D7-iGP (54) background. This cell line (3D7-iGP-*Pf*SPM3-GFP-glmS) allows the robust induction of sexual commitment of the parasite and therefore enables the localization and assessment of *Pf*SPM3 function during gametocytogenesis. As expected, fluorescence microscopy revealed close SPMT localization of *Pf*SPM3 in gametocytes (Fig. 3A). Of note, the *Pf*SPM3-GFP signal disappears earlier than the TubulinTracker signal, indicating loss of detectable *Pf*SPM3 prior to the disassembly of the SPMTs in late gametocyte stages (IV/V gametocytes) (Fig. 3A). Conditional knockdown via the *glmS* system resulted in significantly reduced numbers of gametocytes (mean of 52.6% ± 16.1% at day 10 postinfection) compared to control cultures (Fig. 3B). Gametocytes were morphologically distinct and appeared as malformed (round) and nonfalciform gametocytes. Importantly, these *Pf*SPM3-depleted gametocytes revealed an aberrant SPMT pattern (Fig. 3A and C). To exclude detrimental effects of glucosamine on gametocyte development and SPMT architecture, we assessed the phenotype in the control 3D7-iGP-*Pf*SPM3-GFP parasites with an inactive M9 mutant ribozyme sequence (53). No difference in gametocyte morphology or gametocytemia was observed in these parasites in the presence of 2.5 mM glucosamine (GLCN) (Fig. 3B and C and Fig. S2A). The phenotype was further confirmed by using the 3D7-iGP-*Pf*SPM3-TGD parasite cell line, which expresses the truncated, cytosolic *Pf*SPM3. As for the *glmS*-based approach, after induction of gametocytogenesis the progression and maturation of gametocytes was heavily impaired, resulting in the absence of falciform gametocytes and leading to the formation of malformed, round gametocytes with aberrant SPMT organization (Fig. 3D and E and Fig. S2B). Altogether, these results indicate an essential function of *Pf*SPM3 in gametocyte development and the falciform morphology of P. falciparum gametocytes.

10.1128/mbio.03318-22.2 FIG S2 3D7-iGP-*Pf*SPM3-glmSM9 during gametocyte development. (A) Live-cell microscopy of 3D7-iGP-*Pf*SPM3-GFP-glmSM9 schizonts and stage II to V gametocytes (day 7 to day 13 after gametocyte induction) cultured either with or without (control) 2.5 mM GLCN. Nuclei were stained with Hoechst 33342. Bar, 2 μm. (B) Live-cell microscopy of 3D7-iGP (control) versus 3D7-iGP-*Pf*SPM3-TGD gametocytes (day 5 to day 13 after gametocyte induction). Microtubules were visualized with TubulinTracker, and nuclei were stained with Hoechst 33342. Bar, 2 μm. Download FIG S2, TIF file, 1.8 MB. Copyright © 2023 Wichers-Misterek et al. 2023 Wichers-Misterek et al. https://creativecommons.org/licenses/by/4.0/ This content is distributed under the terms of the Creative Commons Attribution 4.0 International license.

### SPM3 deletion in P. berghei impacts sporozoite motility and transmission by mosquitoes.

**FIG 4:**
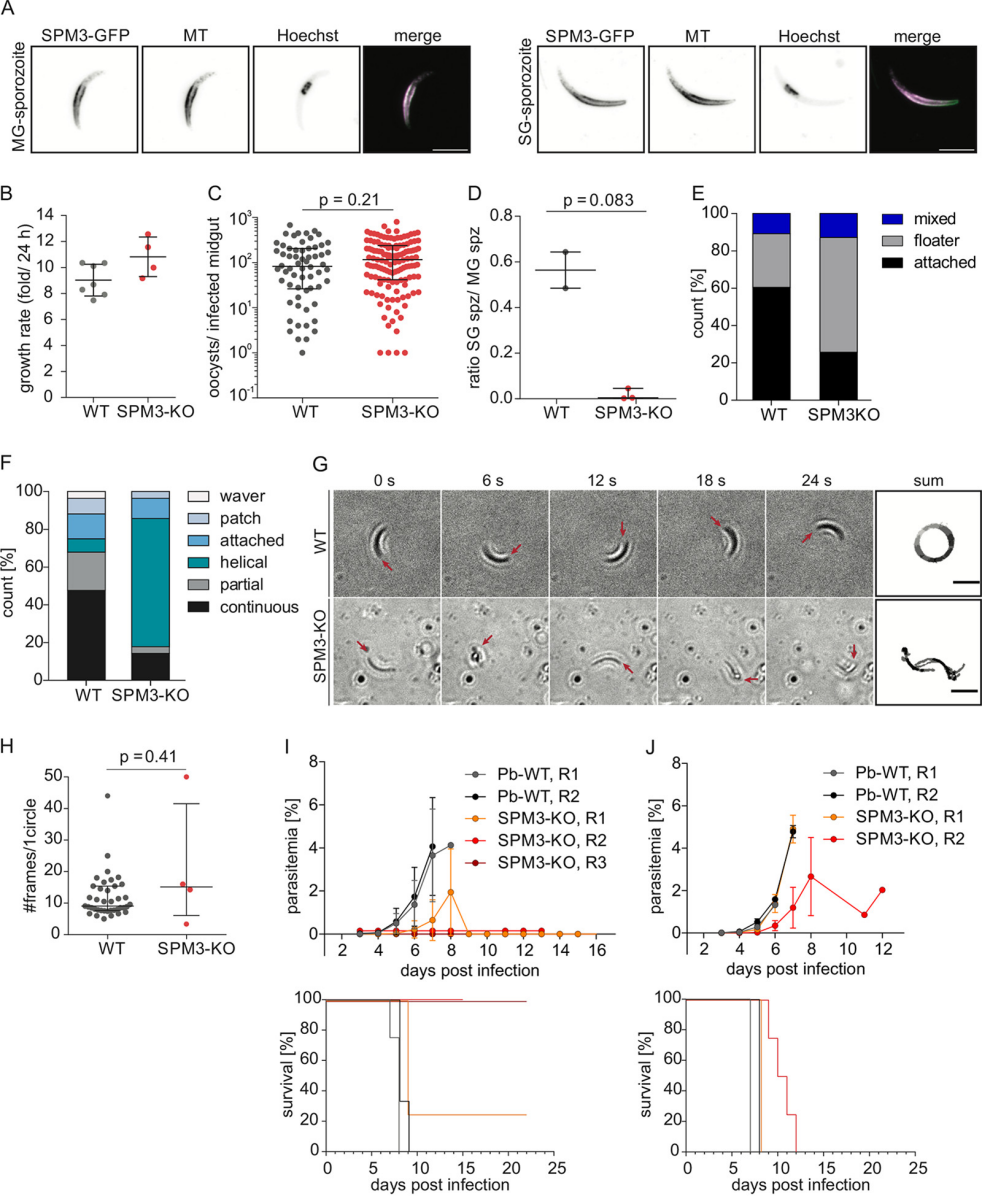
*Pb*SPM3 localizes to SPMTs and is critical for sporozoite motility, salivary gland invasion, and transmission to mammalian hosts. (A) *Pb*SPM3 localizes along microtubules in midgut and salivary gland sporozoites. Representative images of fixed sporozoites. Shown are 2D projections of an acquired Z-stack. MTs were stained with an antitubulin antibody. Nuclei were stained with Hoechst 33342. The merge image shows microtubules in magenta and *Pb*SPM3-GFP in green. Bar, 5 μm. (B) Deletion of *pbspm3* does not affect asexual blood stage growth rate. Asexual blood stage growth rates were calculated based on the parasitemia value at day 8 postinfection with a single infected red blood cell intravenously. PbANKA WT growth rates were plotted as a reference and were determined and previously published by our laboratory (55, 56, 69). Mean fold change within a single parasite replication cycle (24 h for P. berghei) with SD is presented. (C) Similar oocyst numbers and infection rates of wild-type and *Pb*SPM3-KO-infected A. stephensi. Shown are pooled data from two (WT) and three (*Pb*SPM3-KO) independent cage feeds. A total of 79 (WT) and 151 (*Pb*SPM3-KO) mosquitoes were analyzed on day 11 and day 12 postinfection as technical replicates, of which 78.5% (62) and 88.1% (133) were infected, respectively (*P* = 0.08, Fisher’s exact test). For statistical analysis comparing oocyst numbers in infected midguts, a Mann-Whitney test was performed. The black line indicates the median, with error bars representing the IQR. (D) Ratio of salivary gland-resident versus midgut-resident sporozoites determined at day 18 and 20 postinfection from two (WT) and three (*Pb*SPM3-KO) independent cage infections. The *P* value was determined with an unpaired *t* test with Welch’s correction. The black line indicates the median, with error bars representing the IQR. (E) Percentage of wild-type and *Pb*SPM3-KO sporozoites isolated from salivary glands displaying movement after attachment or floating in the medium. Pooled data from two independent cage feeds (WT) with 139 sporozoites and three (*Pb*SPM3-KO) with 109 sporozoites, with two technical replicates per cage infection. For statistical analysis comparing differences in gliding categories, a chi-square test was performed. (F) Different types of movement patterns observed in sporozoites analyzed for panel E. (G) Wild-type-like circular (top) and helical (bottom) movement patterns. Red arrows point to the apical end of the sporozoite. Bar, 10 μm. (H) Sporozoite speeds from continuously gliding wild-type and *Pb*SPM3-KO sporozoites shown in panel F. As the *Pb*SPM3-KO mutant had only four continuously gliding sporozoites in the total sporozoites analyzed from the three biological replicates, only four data points are shown, in comparison to 40 for the wild type. The *P* value was determined with the Mann-Whitney test. The black line indicates the median with the IQR. (I) Parasitemia curves and Kaplan-Meier plots showing increases in blood-stage parasitemia (top) and decrease of mouse survival (bottom) after natural transmission. Two and three independent infections for the wild type and *Pb*SPM3-KO, respectively, using 3 to 4 mice per experiment (totals: WT, *n* = 7; *Pb*SPM3-KO, *n* = 12). See also Table 1. Note that mice from two replicates remained negative over the course of parasitemia monitoring, and hence, the parasitemia curve of replicate 2 was slightly nudged for better visibility. Also, lines in Kaplan-Meier plots in panels J and I were slightly nudged for better visibility. (J) Parasitemia curves and Kaplan-Meier plots showing increases in blood-stage parasitemia (left) and mouse survival after injection of 1,000 sporozoites intravenously. Two independent infections for the wild type (gray and black) and *Pb*SPM3-KO (red and orange) using 3 or 4 mice per experiment (totals: WT, *n* = 7; *Pb*SPM3-KO, *n* = 8).

To extend our functional investigation into the role of SPM3 in mosquito stages, we turned to the rodent model parasite P. berghei, for which the full life cycle is readily available. We first generated a P. berghei parasite line in which the gene ortholog of P. falciparum *spm3* (*Pb*SPM3, PbANKA_1342500) was tagged with GFP (*Pb*SPM3-GFP) (Fig. S3A and B). *Pb*SPM3-GFP was detectable in oocysts, as well as in midgut and salivary gland sporozoites. In sporozoites, the fluorescent signal extended from the apical end toward the rear around the nucleus (Fig. 4A and Fig. S4A). Subsequent costaining with an antitubulin antibody revealed colocalization of *Pb*SPM3-GFP with tubulin (Fig. 4A) in midgut and salivary gland sporozoites. C-terminal tagging of *Pb*SPM3 with GFP did not affect parasite life cycle progression, as SPM3-GFP parasites showed normal midgut infection and midgut oocyst numbers as well as numbers of both midgut and salivary gland sporozoites comparable to those of the wild type (Fig. S4B and C).

10.1128/mbio.03318-22.3 FIG S3 Generation of transgenic P. berghei parasite lines and validation. (A and C) Schematic representation of the KI and KO strategy used to generate *Pb*SPM3-GFP parasites by single-crossover recombination (A) or *Pb*SPM3-KO parasites by double-homologous recombination (B). 3′dhfr, 3′ untranslated region (UTR) of dihydrofolate reductase; ampR, ampicillin resistance; ef1α, elongation factor 1 alpha; tgDHFR, Toxoplasma gondii dihydrofolate reductase; yfcu, yeast cytosine deaminase and uridyl phosphoribosyl transferase. Primers are displayed in the same color as the element they bind to (e.g., P2019 binding to *gfp*). Note that primers in black can bind to both the original and the mutated gene locus. (B and D) Genotyping PCR of the generated *Pb*SPM3-GFP (B) and *Pb*SPM3-KO (D) lines in comparison to the PbANKA wild-type line using primers for 3′ and 5′ integration. Binding sites of the primers used are indicated in panel A. Download FIG S3, TIF file, 0.9 MB. Copyright © 2023 Wichers-Misterek et al. 2023 Wichers-Misterek et al. https://creativecommons.org/licenses/by/4.0/ This content is distributed under the terms of the Creative Commons Attribution 4.0 International license.

10.1128/mbio.03318-22.4 FIG S4 *Pb*SPM3-GFP localization and development of tagged parasite line. (A) Live-cell imaging of endogenously tagged *Pb*SPM3-GFP at the oocyst stage and free midgut (MG-SPZ) and salivary gland sporozoites (SG-SPZ). Bar, 10 μm. (B and C) Endogenous protein tagging has no effect on general parasite development. (B) Similar oocyst numbers of WT and *Pb*SPM3-GFP parasites analyzed on day 11 and day 12 postinfection. Overall, 78.5% (62/79) of mosquito midguts were infected with wild-type parasites compared to 66.7% (22/33) infected with *Pb*SPM3-GFP parasites (*P* = 0.23, Fisher's exact test). For statistical analysis comparing oocyst numbers per infected midgut, a Mann-Whitney test was performed. The black line indicates the median, with error bars representing the interquartile range. (C) Total number of salivary gland-resident versus midgut-resident sporozoites determined at day 18 and 20 postinfection. The black line indicates the median. Note that wild-type data in panels B and C were used for comparison. They represent the wild-type data set shown in Fig. 4C and D. Mosquitoes from two independent wild-type and one *Pb*SPM3-GFP replicate cage infection were analyzed. (D) Knockout of SPM3 leads to dissociation of microtubules from the IMC in midgut sporozoites. Absolute numbers of microtubules close to the IMC (distance of ≤40 nm) in comparison to total microtubule numbers. In total, 31 wild-type sporozoites versus 54 *Pb*SPM3-KO sporozoites were analyzed. The differences in opacity correspond to the number of sporozoites observed with the given microtubule number. Data points with higher opacity correspond to values observed in more sporozoites. The statistical difference in microtubule numbers was determined by a two-sided *t* test comparing differences in numbers of microtubules close to the IMC versus total microtubule numbers between the two parasite lines. (E) Association of microtubules with membranes in midgut sporozoites. Absolute numbers of microtubules close to the membrane (distance of ≤40 nm) in comparison to total microtubule numbers. A total of 11 wild-type sporozoites versus 45 *Pb*SPM3-KO sporozoites were analyzed. The differences in opacity correspond to the number of sporozoites observed with the given microtubule number. Data points with higher opacity correspond to values observed in more sporozoites. The statistical difference in microtubule numbers was determined by a two-sided *t* test comparing differences in numbers of microtubules close to the membrane versus total microtubule numbers between the two parasite lines. (F) Distance between microtubules and membrane in midgut sporozoites, with the black line indicating the median. The dashed line indicates the cutoff value of 40 nm; microtubules at distances of 40 nm or less were defined as close. The grey background highlights all values not considered close to the membrane, with the corresponding percentages above. A total number of 107 wild-type and 503 *Pb*SPM3-KO microtubules was measured. The spread of microtubule-membrane distances was statistically analyzed using a linear mixed model. Download FIG S4, TIF file, 1.0 MB. Copyright © 2023 Wichers-Misterek et al. 2023 Wichers-Misterek et al. https://creativecommons.org/licenses/by/4.0/ This content is distributed under the terms of the Creative Commons Attribution 4.0 International license.

In order to probe into *Pb*SPM3 function, we generated a clonal *Pb*SPM3 knockout (*Pb*SPM3-KO) cell line (Fig. S3C and D). Similar to the P. falciparum gene disruption, no apparent difference in blood-stage growth was detected for *Pb*SPM3-KO compared to our previously determined growth rates of P. berghei wild-type parasites (55, 56) (Fig. 4B). Next, to investigate the progression of this parasite line in mosquitoes, we fed naive Anopheles stephensi mosquitoes on mice infected with *Pb*SPM3-KO parasites. Surprisingly, we found that infection and colonization of the mosquito midgut by *Pb*SPM3-KO parasites were similar to those by wild-type parasites, as measured by the percentage of fed mosquitoes that had oocysts and the median numbers of oocysts per infected mosquito (*Pb*SPM3-KO, 116; interquartile range [IQR], 42 to 238; WT, 83; IQR, 26 to 206) (Fig. 4C). Furthermore, we found numbers of *Pb*SPM3-KO sporozoites (median, 83,000; IQR, 49,000 to 94,000) similar to those of the wild type (median, 39,000; IQR, 30,000 to 63,000) maturing in midgut oocysts (Table 1). These findings indicate that neither gametocyte development, exflagellation, ookinete development, nor midgut traversal is significantly affected by *Pb*SPM3 gene knockout.

**TABLE 1:** Numbers and infectivity of *Pb*SPM3-KO sporozoites compared to the WT^*a*^

Parasite line	No. of sporozoites/mosquito (Mean ± SD) (replicates)		Infection with 1,000 sporozoites i.v.		Natural infection (by bite)	
	Midgut	Salivary gland	Prepatency time (days) (no. infected/total)	% parasitemia at day 6 (infected only)	Prepatency time (days) (no. infected/total)	% parasitemia at day 6 (infected only)
WT	34,000 ± 6,000	21,000 ± 1,000	3.6 (7/7)	1.4	3.7 (7/7)	1.5
	55,000 ± 16,000	27,000 ± 10,000				
*Pb*SPM3-KO	70,000 ± 22,000	3,000 ± 1,000	4.6 (8/8)	0.8	5.7 (3/12)	0.3
	90,000 ± 9,000	200 ± 40				
	67,000 ± 18,000	300 ± 50				

a *n* = 2 for WT, *n* = 3 for *Pb*SPM3-KO transmitted by bite, and *n* = 2 for *Pb*SPM3-KO transmitted by intravenous (i.v.) injection. Parasitemia values are given for all mice that became positive within 20 days; C57/BL6 mice were used.

Next, we tested whether *Pb*SPM3-KO might affect the further development of sporozoites. While salivary glands of mosquitoes infected with wild-type parasites showed over 20,000 sporozoites (median, 21,000; IQR, 18,000 to 34,000), only a few hundred were found for *Pb*SPM3-KO sporozoites (median, 300; IQR, 220 to 2,690), indicating a drastic (99%) decrease in the ratio of salivary gland sporozoites to midgut sporozoites (Fig. 4D; Table 1).

To pinpoint the cause for the reduced number of *Pb*SPM3-KO sporozoites in salivary glands, we assessed the effect of *Pb*SPM3 deletion on sporozoite motility, an essential feature for the parasite entry into salivary glands and for establishing infection in human liver cells. Wild-type P. berghei sporozoites isolated from salivary glands attach and migrate on flat surfaces in a counterclockwise circular manner at speeds exceeding 1 μm/s (40). While 60% of the wild-type sporozoites attached and moved on the glass coverslip, most *Pb*SPM3-KO sporozoites (61%) remained floating in the medium (Fig. 4E). For the majority of *Pb*SPM3-KO sporozoites that managed to attach and move, a striking new phenotype of sporozoite locomotion was revealed: the parasites moved in a helical way (Fig. 4F and G and Movies S1 to 3) and deviated from the circular path observed in most of the wild-type sporozoites. The few *Pb*SPM3-KO sporozoites that showed persistent circular gliding motility moved with a speed similar to that of wild-type sporozoites (Fig. 4H).

10.1128/mbio.03318-22.6 MOVIE S1 Wild-type sporozoite gliding motility. Individual sporozoite moving in a glass-bottom 96-well plate imaged with a 25× objective, with one frame every 3 s over 100 frames. Download Movie S1, AVI file, 1.7 MB. Copyright © 2023 Wichers-Misterek et al. 2023 Wichers-Misterek et al. https://creativecommons.org/licenses/by/4.0/ This content is distributed under the terms of the Creative Commons Attribution 4.0 International license.

10.1128/mbio.03318-22.7 MOVIE S2 PbSPM3-KO sporozoite aberrant gliding motility, example A. Individual sporozoite moving in a glass-bottom 96-well plate imaged with a 25× objective with one frame every 3 s over 100 frames. Download Movie S2, AVI file, 1.4 MB. Copyright © 2023 Wichers-Misterek et al. 2023 Wichers-Misterek et al. https://creativecommons.org/licenses/by/4.0/ This content is distributed under the terms of the Creative Commons Attribution 4.0 International license.

10.1128/mbio.03318-22.8 MOVIE S3 PbSPM3-KO sporozoite aberrant gliding motility, example B. Individual sporozoite moving in a glass-bottom 96-well plate imaged with a 25× objective with one frame every 3s over 100 frames. Download Movie S3, AVI file, 1.5 MB. Copyright © 2023 Wichers-Misterek et al. 2023 Wichers-Misterek et al. https://creativecommons.org/licenses/by/4.0/ This content is distributed under the terms of the Creative Commons Attribution 4.0 International license.

To further dissect the physiological consequences of this aberrant cell motility, we investigated the transmission capacity of mosquitoes infected with *Pb*SPM3-KO parasites. We found that only 3 of 12 mice became infected after being bitten by *Pb*SPM3-KO infected mosquitoes, while all mice bitten by mosquitoes infected with wild-type parasites became infected (Fig. 4I; Table 1). Furthermore, we noticed a delay in appearance (prepatency) of 2 days in mice that did become infected with *Pb*SPM3-KO parasites. This drop in infectivity is likely due to the much lower load of sporozoites within the salivary glands. We next tested mouse liver infectivity of *Pb*SPM3-KO sporozoites by direct intravenous inoculation of an equal number of purified sporozoites, as the lower level of sporozoites in salivary glands made direct transmission from mosquito to mice unreliable for this assay (57). All mice infected through the intravenous injection of 1,000 *Pb*SPM3-KO sporozoites became infected (Fig. 4J). These results suggest that the reduced number of *Pb*SPM3-KO sporozoites in the salivary glands and their aberrant motility affect the transmission from the mosquito to the mammalian host, but not the invasion of liver cells.

### *Pb*SPM3 deletion disturbs SPMT architecture in P. berghei.

**FIG 5:**
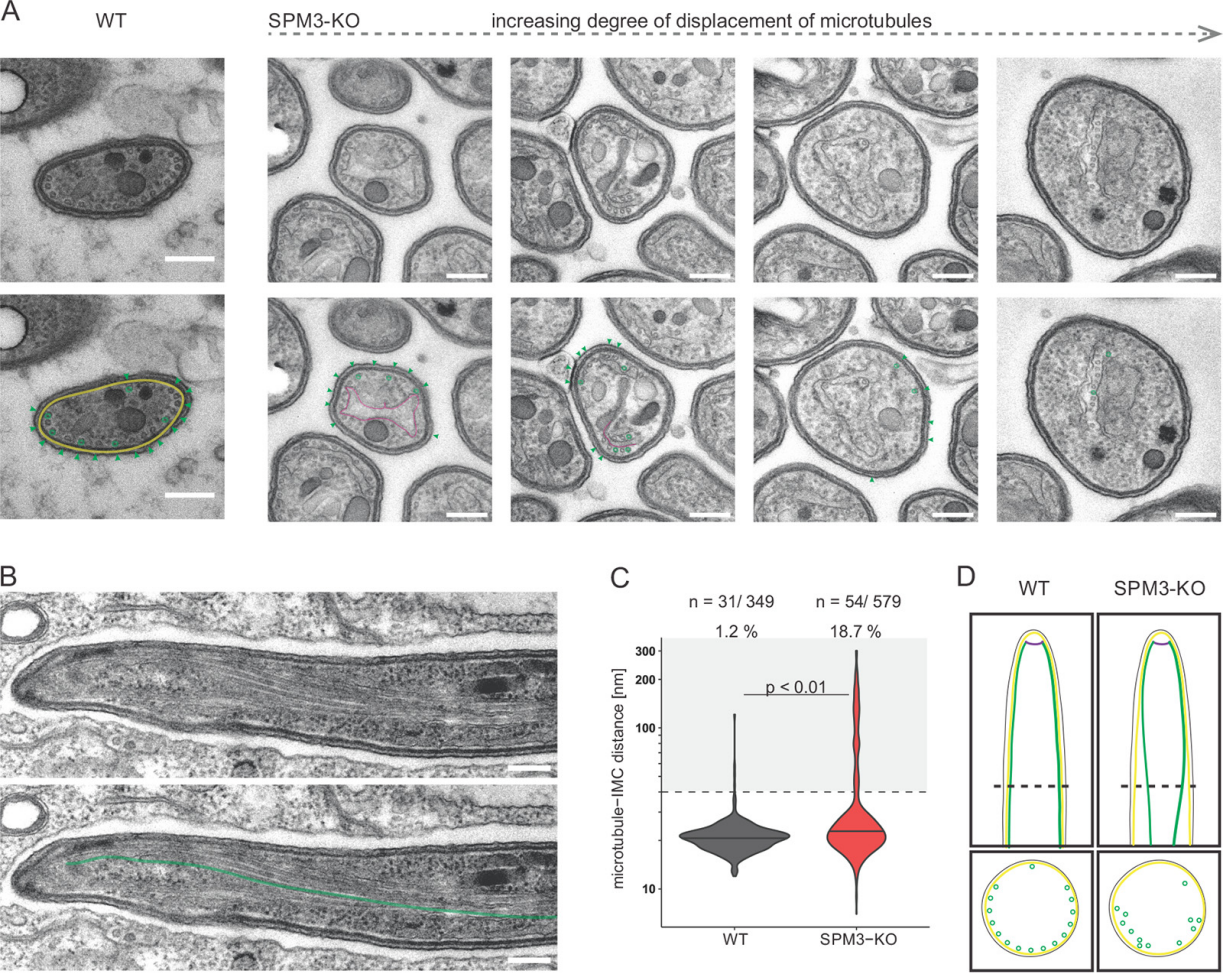
SPMTs dissociate from the IMC in *Pb*SPM3-KO sporozoites. (A) Transmission electron micrographs showing cross sections through WT (left) and *Pb*SPM3-KO (right) P. berghei sporozoites within oocysts at day 15 after mosquito infection. Single images are ordered according to increasing degree of microtubule displacement. Bar, 200 nm. Green, SPMTs; yellow, IMC; magenta, membrane of unknown origin. (B) Longitudinal section through a *Pb*SPM3-KO midgut sporozoite. Note the dissociation of the microtubules from the IMC with increasing distance from the apical end. (C) Distance between microtubules and IMC, with the black line indicating the median. The dashed line indicates the preset cutoff value of 40 nm; distances of 40 nm or less were taken as representing microtubules close to the IMC, according to the findings in microtubule-IMC distance by Ferreira et al. (26). The gray background highlights all values not considered close to the IMC, with the corresponding percentages above. In total, 349 wild-type and 579 *Pb*SPM3-KO microtubules were measured. Spread of microtubule-IMC distance was statistically analyzed using a linear mixed model. (D) Proposed model showing the dissociation of the microtubules from the IMC with increasing distance from the apical end in the *Pb*SPM3-KO. The dashed line in the longitudinal section shows the position of the cross section pictured below it.

To identify the cause of the aberrant motility of *Pb*SPM3-KO sporozoites, we investigated the SPMT organization in fixed midgut sporozoites by transmission electron microscopy (TEM). Preparation of thin sections from oocysts allows the imaging of sporozoites in cross sections (Fig. 5A). We found that near the apical end of *Pb*SPM3-KO sporozoites, the arrangement of SPMTs largely followed that of wild-type sporozoites, with a single SPMT being somewhat apart from the others and all SPMTs being closely associated with the inner membrane complex (Fig. 5A). However, we found that further from the apical end (as discernible by an increased sporozoite diameter, the presence of apical organelles such as micronemes and rhoptries, and an increased intermicrotubule distance), the SPMTs were not associated with the IMC and often appeared deep within the cytoplasm (Fig. 5A and B and Fig. S5A to C). This suggests a dissociation from the IMC. We next quantified the distance between SPMTs and IMC by measuring from microtubule center toward the inner side of the IMC. Also, the SPMT-IMC distance was increased in the SPM3-KO parasites, with only 1% of SPMTs being more than 40 nm apart from the IMC in the wild type compared to 19% in the *Pb*SPM3-KO mutants (Fig. 5C; Fig. S4D). We also observed SPMTs associated with non-IMC membranes in the cytoplasm for both WT and SPM3-KO parasites (Fig. 5A, magenta). There was no difference between wild-type and SPM3-KO parasites in the number of SPMTs associated with these membranes or the distance between SPMTs and the membranes (Fig. S4E and F). Taken together, these observations point to a role of *Pb*SPM3 in anchoring the SPMT to the IMC in sporozoites.

10.1128/mbio.03318-22.5 FIG S5 (A and B) Transmission electron micrographs showing cross sections through *Pb*SPM3-KO (right) P. berghei sporozoites within oocysts at day 15 after mosquito infection. Note the different phenotypes in individual sporozoites, with some cells displaying an MT distribution similar to that of wild-type sporozoites. Bars, 500 nm (A) and 200 nm (B). (C) Longitudinal section through a *Pb*SPM3-KO midgut sporozoite. Note the dissociation of the microtubules from the IMC. Bar, 500 nm. Download FIG S5, TIF file, 2.4 MB. Copyright © 2023 Wichers-Misterek et al. 2023 Wichers-Misterek et al. https://creativecommons.org/licenses/by/4.0/ This content is distributed under the terms of the Creative Commons Attribution 4.0 International license.

## DISCUSSION

In this study, we identified SPM3, a novel *Plasmodium*-specific microtubule-associated protein critical for gametocyte development and morphology of P. falciparum gametocytes and motility of P. berghei sporozoites. *Pf*SPM3 is a high-molecular-weight protein with no predicted transmembrane domain, initially identified as a potential IMC component by *Pf*PhIL1-based proximity-dependent biotinylation in schizonts (41) and by *Pf*BLEB-based proximity-dependent biotinylation in stage II-III gametocytes, a protein (*Pf*BLEB) associated with the basal complex of the parasite (58). By using colocalization approaches, we showed that *Pf*SPM3 colocalizes with the SPMTs in asexual and sexual stages and only partially with the IMC compartment. In asexual stages, *Pf*SPM3 appears to be more pronounced at one pole of the SPMTs, in contrast to *Pf*SPM1, a homologue of a well-characterized T. gondii MAP, which showed a homogenous pattern along the microtubule. MAPs display restricted localization patterns along the SPMTs and may compartmentalize different regions of the microtubule by interacting with pellicular structures such as the IMC (32). Of note, similar to the pronounced apical localization observed for *Pf*SPM3, *Tg*TLAP3 has been shown to selectively coat the SPMTs in the apical cap region and the intraconoidal microtubules of both mother and daughter cells (32).

*Pf*SPM3 appears to be—in agreement with genome-wide mutagenesis screens (59, 60)—dispensable for asexual blood stage proliferation, since depletion of the protein showed no growth effect, indicating redundancy of MAPs or a stage-specific function. In accordance with the latter, we provide experimental evidence that SPM3 is a critical factor for gametocyte development and morphology in P. falciparum. Depletion of *Pf*SPM3 led to an aberrant organization of the SPMTs in gametocytes, a reduction in the number of gametocytes, and the formation of round gametocytes differing from the typical falciform shape of P. falciparum.

The drastic morphological transformation observed during gametocytogenesis from a committed ameboid trophozoite to a banana-shaped, falciform gametocyte is orchestrated by the expansion of the IMC network and the extension of SPMT arrays (22). These pellicular components maintain the elongated shape of P. falciparum and other *Laverania* gametocytes (24). SPMTs also confer rigidity to the immature gametocytes, which is proposed to be involved in the sequestration of gametocytes in the microvasculature and tissues of the human host to avoid splenic clearance (25). Later depolymerization and disassembly of the SPMT are expected to increase cellular deformability and release into the bloodstream, where gametocytes are taken up by the feeding mosquitoes. The falciform morphology is the hallmark of *Plasmodium* species within the clade *Laverania* and is expected to favor the transmigration from mature gametocytes from the bone marrow reservoir to the bloodstream (22, 24). Nevertheless, the molecular components that determine this morphology remain largely unknown.

The phenotype observed in gametocytes upon depletion of *Pf*SPM3 and the low conservation of the protein outside the subgenus *Laverania* could indicate that *Laverania* SPM3 proteins are somehow involved in the falciform architecture of the late-stage gametocytes. Consistent with this notion, we observed no evident phenotype in the roundish P. berghei gametocytes from rodents. Previous studies with nonfalciform round gametocytes of P. berghei and P. knowlesi show a discontinuous IMC network and pellicle architecture that does not surround these stages (61). This reflects the pronounced structural differences in gametocyte development between the rodent-infecting P. berghei and the human-infecting P. falciparum parasites (25). The stage-specific phenotypes observed in P. berghei and P. falciparum indicate that in the different species, MAPs can be repurposed according to requirements of the different stages and might be explained by the limited conservation outside the clade *Laverania*. However, the phenotype in P. falciparum SPM3-depleted gametocytes and mosquito stages could not be further assessed in this study due to the intrinsic deficiency in the ability of the 3D7 strain to exflagellate *in vitro* and to develop in the mosquito host. Further studies with NF54 would be necessary to elucidate the role in mosquito stages, but such investigations are beyond the scope of this study.

Nevertheless, to investigate the role of SPM3 in mosquito stages, we switched to the malaria mouse model. There, P. berghei sporozoites showed a severe defect in motility and the ability to enter salivary glands and consequently a lower transmission efficiency. This phenotype could be explained by the impaired SPMT organization due to the lack of microtubule anchoring to the IMC (Fig. 5D). Microtubules in sporozoites are organized in a polar fashion, with all the microtubules underlying the IMC and all but one extending over two-thirds of the circumference and a single microtubule being located in the remaining third (62). How this asymmetry is formed is not clear, but it has been speculated that this polarity contributes to the counterclockwise continuous circular movement of *Plasmodium* sporozoites (40, 63). This suggests that displacement of microtubules from the IMC could affect persistent circular motility, as we found here for *Pb*SPM3-KO sporozoites (Fig. 4E to G). In line with this, the observed helical movement is reminiscent of the motion observed for P. berghei ookinetes and T. gondii tachyzoites (64, 65), where the microtubules are equally spaced around the circumference.

Curiously, the deletion of SPM3 causes a problem for P. berghei sporozoites but not ookinetes, while SPM3 is expressed in both stages (66, 67). We found previously that mutations in either actin or actin-associated proteins that were expressed in both ookinetes and sporozoites invariably caused problems for sporozoites and not ookinetes (68–70). Those differences could arise due to the different requirement for speed and force generation by these parasite forms. Likely, the sporozoites are more susceptible to subtle changes. In keeping with this line of thought, one possible explanation for an absence of an SPM3-KO phenotype in ookinetes could be their larger number of microtubules (around 50 compared to the sporozoites’ 16) (7, 26, 62, 71) and their lower speed as they penetrate the midgut (72–74). Additionally, or alternatively, there might be a redundant protein expressed in ookinetes that is absent from sporozoites. More work will be required to understand these intriguing processes in more detail.

MAPs influence several properties and define individual subpopulations of SPMT in the different stages of apicomplexan parasites. The exact role of SPM3 in microtubule organization and pellicle architecture remains to be elucidated. The close association of the SPMTs with the cytosolic face of the parasite pellicle is expected to be mediated by MAPs (11, 40, 75). We can speculate that in P. falciparum, SPM3 in gametocytes provides specialized contact sites between SPMTs and the IMC that support SPMT organization, cell elongation, and ultimately falciform cell shape. Consequently, loss of SPM3 may disrupt the interaction with IMC components such as PhIL1, alveolins, and other IMC proteins, leading to SPMT disorganization and rounding of the cell. Additional *Pf*SPM3-specific interaction partners in gametocytes remain to be identified. In accordance, our ultrastructural analysis in P. berghei sporozoites suggests that *Pb*SPM3 might connect the SPMTs to the IMC. Notably, the function of SPM3 appears to be early in gametocyte development, since the *Pf*SPM3 signal vanishes prior to the disassembly of SPMTs in stage IV/V gametocytes. Association of *Pf*SPM3 to the SPMT may also influence stability and depolymerization of SPMT as shown for T. gondii SPM1, TLAP2, and TLAP3, MAPs that protect the stability of the SPMT in a region-dependent manner (32).

In conclusion, we identified here a *Plasmodium*-specific microtubule-associated protein that has essential functions for gametocyte and sporozoite development and hence is critical for malaria transmission.

## MATERIALS AND METHODS

### P. falciparum culture.

Blood stages of P. falciparum 3D7 (76) were cultured in human red blood cells (O^+^ or B^+^; Blood Bank, Universitätsklinikum Hamburg-Eppendorf). Cultures were maintained at 37°C in an atmosphere of 1% O_2_, 5% CO_2_, and 94% N_2_ using RPMI complete medium containing 0.5% Albumax according to standard protocols (77). In order to obtain highly synchronous parasite cultures, late schizonts were isolated by Percoll gradient centrifugation (78) and cultured with fresh erythrocytes for 4 h. Afterwards, sorbitol synchronization (79) was applied in order to remove remaining schizonts, resulting in a highly synchronous ring-stage parasite culture with a 4-h age window.

Induction of gametocytogenesis was done as previously described (54, 80). Briefly, GDV1-GFP-DD expression was achieved by addition of 2 or 4 μM Shield-1 to the culture medium, and gametocyte cultures were treated with 50 mM *N*-acetyl-d-glucosamine (GlcNAc) for 5 days starting 72 h after Shield-1 addition to eliminate asexual parasites (81). Alternatively, asexual ring-stage cultures with >10% parasitemia, cultured in the presence of choline, were synchronized with sorbitol (79) and washed twice in choline-free RPMI medium. Cells were kept in choline-free medium for the entirety of the assay. After one reinvasion cycle, cultures at the trophozoite stage were treated with 50 mM GlcNAc (81) and kept on this for 5 days. Gametocytes were maintained in RPMI complete medium containing 0.25% Albumax and 0.25% sterile filtered human serum (Interstate Blood Bank, Inc., Memphis, TN, USA).

### Cloning of plasmid constructs for parasite transfection.

For endogenous tagging of *Pf*SPM3 (PF3D7_1327300) using the SLI system (42) and *glmS*-based conditional knockdown (53, 82) a 1,021-bp homology region was amplified using 3D7 genomic DNA (gDNA) and cloned into pSLI-PMRT1-GFP-glmS (83) using NotI and MluI restriction sites.

For SLI-based targeted gene disruption (SLI-TGD) of *Pf*SPM3, a 399-bp homology region was amplified using 3D7 gDNA and cloned into the pSLI-TGD plasmid (42) using NotI and MluI restriction sites.

For overexpression constructs, the full-length sequences of PhIL1 (PF3D7_0109000), SPM1 (PF3D7_0909500), and PF3D7_1345600 were amplified from parasite cDNA and cloned into pARL-*^ama1^*AIP-mCherry-yDHODH (41) using the XhoI and KpnI restriction sites. Oligonucleotides used to generate the DNA fragments are summarized in Table S1.

10.1128/mbio.03318-22.9 TABLE S1 Oligonucleotides used for cloning and diagnostic genotyping PCR. Download Table S1, PDF file, 0.03 MB. Copyright © 2023 Wichers-Misterek et al. 2023 Wichers-Misterek et al. https://creativecommons.org/licenses/by/4.0/ This content is distributed under the terms of the Creative Commons Attribution 4.0 International license.

For colocalization experiments, the plasmids pARL-*^ama1^*ALV5-mCherry (41) and pARL-*^ama1^*ARO-mCherry (41) were used.

For gene deletion of *Pb*SPM3 (PBANKA_1342500), both a 3′ and a 5′ homology region (750 and 717 bp) were amplified from *Pb*ANKA wild-type-genomic DNA and cloned into the Pb262 vector (84) using HindIII/XhoI and EcoRI/EcoRV restriction sites. The Pb262 vector contains the *hDHFR* gene, which allows for positive selection using the drug pyrimethamine. Prior to transfection, the vector was linearized using SacII and PmeI followed by ethanol precipitation.

For endogenous tagging of *Pb*SPM3 (PBANKA_1342500), a 992-bp 3′ homology region was amplified from PbANKA wild-type genomic DNA. The reverse primer encoded the linker sequence that was used for GFP tagging of *Pf*SPM3, as described by Birnbaum et al. (42). The amplicon was cloned into the pL18 vector (85) using EcoRI/BamHI restriction sites. Prior to transfection, the vector was linearized using the SwaI restriction enzyme followed by ethanol precipitation.

All oligonucleotides used to generate DNA fragments as well as those used for genotyping PCRs are listed in Table S1.

### Transfection of P. falciparum.

For transfection, Percoll-purified (78) parasites at the late schizont stage were transfected with 50 μg plasmid DNA using Amaxa Nucleofector 2b (Lonza, Switzerland) as previously described (86). Transfectants were selected using either 4 nM WR99210 (Jacobus Pharmaceuticals) or 0.9 μM DSM1 (87) (BEI Resources). In order to select for parasites carrying the genomic modification via the SLI system (42), G418 (Thermo Fisher, USA) at a final concentration of 400 μg/mL was added to a culture with about 5% parasitemia. The selection process and integration test were performed as previously described (42).

### Generation of P. berghei parasite lines.

The linearized *Pb*SPM3-KO vector was transfected into the unmodified *P. berghei* strain ANKA using standard protocols (88). Parasites that integrated the desired DNA construct were selected by administration of pyrimethamine (0.07 mg/mL) via the mice’s drinking water. Parasites from transfections are mixed populations, and hence, a limiting dilution was performed to isolate clonal lines. For this, a single blood-stage parasite was injected into each of 8 Swiss mice intravenously. Once mice reached 1 to 3% parasitemia, blood was collected via cardiac puncture from anesthetized mice (100 mg/kg ketamine and 3 mg/kg xylazine; Sigma-Aldrich). Whole-blood aliquots were stored in liquid nitrogen. Genomic DNA was isolated from whole blood and tested for correct construct integration by PCR. Erythrocytes were lysed in 1.5 mL phosphate-buffered saline (PBS) containing 0.03% saponin. After centrifugation and washing, the genomic DNA was isolated using a blood and tissue kit (Qiagen Ltd.) according to the manufacturer’s protocol. All generated isogenic parasite lines were analyzed via PCR. Parasite lines with the correct genotype were assumed to be isogenic.

### Determination of P. berghei asexual blood stage growth rate.

Blood smears from all mice used for limiting dilution and carrying an isogenic parasite line were done. The parasitemia determined on day 8 postinfection was used to back-calculate the growth rate, as described elsewhere (89, 90).

### Mosquito infection.

Frozen parasite stocks were thawed and injected intraperitoneally (100 to 150 μL) into one mouse. Parasites were allowed to grow for 4 to 6 days, with the infection rate being monitored by blood smears. Once the infected mouse reached 2 to 3% parasitemia, the mouse was anesthetized (100 mg/kg ketamine and 3 mg/kg xylazine; Sigma-Aldrich) and bled by cardiac puncture. From this donor mouse, 20 million parasites were transferred into two naive recipient mice by intraperitoneal injection. Three days after fresh blood transfer, exflagellation of parasites was checked first; mice were anesthetized (120 mg/kg ketamine and 16 mg/mL xylazine) and then placed in a cage containing around 200 female Anopheles stephensi mosquitoes, which had been starved for 3 to 5 h by removal of sugar and salt pads. Mosquito were allowed to feed on mice for at least 15 min up to 30 min. Infected mosquitos were kept at 21°C and 70% humidity.

### Exflagellation assay *in vitro*.

A drop of tail blood from an infected mouse was placed onto a glass slide, tightly covered with a cover glass, and placed at 21°C for 12 min. Exflagellation centers were observed by light microscopy at ×40 magnification with a phase-contrast ring on a Zeiss Axiostar microscope. The number of exflagellation centers was roughly counted in several fields with comparable erythrocyte densities.

### Analysis of oocyst development by mercurochrome staining.

Midguts of infected mosquito were isolated on day 11 and day 12 after a mosquito blood meal from three independent mosquito feeds. A minimum of 50 mosquitos per feed replicate were dissected. Midguts were dissected in 100 μL of PBS and permeabilized with 1% Nonidet P40 in PBS (AppliChem GmbH) for 20 min at room temperature (RT). The supernatant was removed, and midguts were stained for 30 min to 2 h in 0.1% mercurochrome in PBS (NF XII; Sigma-Aldrich). The staining solution was discarded, and midguts were washed several times with PBS until the supernatant became clear. Stained midguts were transferred onto a glass slide, leaving some PBS on top, and sealed with a coverslip. Midguts were imaged using an epifluorescence microscope (CellObserver; Zeiss) using a 10× (numerical aperture [NA], 0.5; air) objective with a green filter, and both the infection rate and the number of oocysts were determined.

### Sporozoite isolation, counting, and imaging.

Midgut and salivary gland sporozoites of infected mosquitoes were isolated on day 18 and day 20 after a mosquito blood meal from three independent mosquito feeds. A minimum of 10 mosquitoes was dissected per count. The midguts and salivary glands were dissected on ice in RPMI and crushed with a pestle before counting sporozoites using a Neubauer counting chamber. For imaging, the solution containing crushed salivary glands was filled up to 1 mL with RPMI, and was carefully underlaid with 3 mL of 17% Accudenz. Centrifugation at 2,800 rpm for 20 min at room temperature separated sporozoites from cell debris. The sporozoite-containing interphase was collected (total volume of 1.4 mL), and sporozoites were pelleted for 3 min at 1,000 rpm (Biofuge Primo; Thermo Fisher Scientific). The supernatant was removed; RPMI containing 3% bovine serum albumin (Carl Roth) was added to the sporozoites, and the resulting mixtures were transferred into the wells of an optical-bottom 96-well plate (Thermo Fisher Scientific). The plate was centrifuged for 3 min at 1,000 rpm (Multifuge S1-R; Heraeus) and imaged using an epifluorescence microscope (CellObserver; Zeiss). Movies were obtained using a 25× (NA, 0.8; water) objective at a speed of 1 frame every 3 s with 100 cycles imaged per movie. Movies were taken until 1 h after sporozoite activation. Videos were analyzed for 5 min with Fiji (version 2.0.0 rc 64/1.51s) (91). Gliding assays were carried out as technical replicates on day 19 and day 22 for each cage infection. For analysis, data from two (WT) and three (*Pb*SPM3-KO) biological replicates were pooled and analyzed in a single-blind fashion. Gliding motility was categorized into different patterns as described before (92). Briefly, sporozoites’ motility behavior can be distinguished as follows: continuous (persistently moving sporozoites for at least 50 of 100 frames without stopping more than 10 frames in a row), partial (sporozoites moving in a circular manner but not persistently), helical (sporozoites moving in a new type of motility as illustrated in Fig. 4E and F), attached (sporozoites adhering to the substrate but showing no sign of movement), patch (sporozoites moving actively over a single adhesion spot in a back-and-forth manner), wavers (sporozoites attached with one end and the other end moving actively through the medium), and floating (sporozoites floating in the medium without contact with the substrate).

### Mouse infection by natural transmission and intravenous injection of sporozoites.

For natural transmission via mosquito bite, 10 female mosquitos that had had their blood meal 21 days before the experiment were transferred into cups the day before the experiment. Mosquitoes were starved for 3 to 5 h the next day by removal of sugar and salt pads. Naive C57/BL6 mice were anaesthetized with 120 mg/kg ketamine and 16 mg/mL xylazine, and one mouse was placed per cup, allowing mosquitoes to feed for at least 15 min up to 30 min with a minimum number of 6 mosquitos having fed. To confirm that mosquitos were infected, salivary glands of all blood-fed mosquitos were dissected as described above, and sporozoite numbers were determined.

For intravenous injection of sporozoites, salivary gland sporozoites were dissected as described above, and 1,000 sporozoites (in 100 μL PBS) were injected into the lateral tail vein of a naive C57/BL6 mouse.

Daily blood smears were taken from day 3 postinfection at approximately the same time as the infection experiment. Once the infected mice reached 2 to 3% parasitemia, they were sacrificed by cervical dislocation. Mice that did not become positive 20 days postinfection were considered negative.

### Fluorescence imaging of fixed P. berghei sporozoites.

Microtubules and SPM3 localization of fixed Plasmodium berghei sporozoites were visualized using a Zeiss Airyscan 2 LSM900 laser scanning confocal microscope. Both midgut and salivary gland sporozoites were isolated as described above but without subsequent Accudenz purification. Instead, sporozoites were directly pelleted for 3 min at 13,000 rpm (Biofuge Primo; Thermo Fisher Scientific). The supernatant was removed, and sporozoites were resuspended in RPMI containing 3% bovine serum albumin (Carl Roth). For imaging, 30,000 to 50,000 sporozoites were transferred into a well of a Labtek slide (μ-slide, 8-well ibiTreat, no. 80826). Sporozoites were pelleted for 3 to 4 min at 800 rpm (Multifuge S1-R; Heraeus) and then incubated for 10 min at room temperature, allowing sporozoites to attach and start gliding while leaving the supernatant on top. Sporozoites were washed three times with RPMI before being fixed in 4% paraformaldehyde (PFA) in PBS at room temperature. After 1 h of incubation, fixative was removed by washing sporozoites three times with RPMI followed by permeabilization with 0.5% Triton X-100 in PBS for 1 h at room temperature. Before incubation with primary antibody, sporozoites were again washed three times with RPMI. Sporozoites were incubated for 1 h at room temperature with mouse antitubulin (1:500; Sigma-Aldrich, no. T5168) to stain microtubules, followed by three washing steps with RPMI. Alexa Fluor 546-conjugated goat anti-mouse immunoglobulin (1:500; Invitrogen, no. A11030) was used as the secondary antibody and incubated for 30 min up to 1 h at RT before incubation of sporozoites for 15 min in RPMI containing 1:1,000-diluted Hoechst 33342 from a 10 μM Hoechst 33342 stock solution (Thermo Fisher Scientific). Sporozoites were washed three times in RPMI and subsequently imaged. Z-stacks were acquired with a z-spacing of 0.5 μm and a total of 29 layers being taken. Images were directly 3D processed by the internal 3D Airyscan processing option.

### Fluorescence imaging of live P. berghei parasites.

Midguts were isolated as before and transferred onto a glass slide, leaving some PBS on top, and sealed with a coverslip. Sporozoites were activated in 3% BSA in RPMI and transferred to an optical 96-well plate (Thermo Fisher Scientific). The plate was centrifuged for 3 min at 1,000 rpm (Multifuge S1-R; Heraeus) and imaged using an epifluorescence microscope (CellObserver; Zeiss). Images from both oocysts and sporozoites were obtained using a 63× (NA, 1.4; oil) objective with an exposure of 80 milliseconds.

### TEM.

Highly infected midguts were fixed by incubation in 4% paraformaldehyde and 4% glutaraldehyde diluted in 100 mM sodium cacodylate buffer at 4°C overnight. Fixed samples were washed three times in 100 mM sodium cacodylate buffer at RT for 5 min. A secondary fixation was done in 1% osmium (in 100 mM sodium cacodylate buffer) at RT for 60 min. Samples were washed twice with 100 mM sodium cacodylate buffer and twice in double-distilled water (ddH_2_O) and then contrasted with 1% uranyl acetate (in ddH_2_O) at 4°C overnight. Samples were washed twice with ddH_2_O for 10 min and then dehydrated by incubating in increasing concentrations of acetone (30%, 50%, 70%, and 90%) for 10 min and twice in 100% acetone for 10 min. Samples were adapted to Spurr’s solution (23.6% epoxycyclohexylmethyl-3,4-epoxycyclohexylcarboxylate [ERL], 14.2% ERL-4206 plasticizer, 61.3% nonenylsuccinic anhydride, 0.9% dimethylethanolamine) by incubating in increasing concentrations (25%, 50%, and 75%) at RT for 45 min and at 100% at 4°C overnight. Midguts were resin embedded with Spurr’s solution at 60°C overnight. Embedded midguts were trimmed, and 70-nm-thick sections were imaged on a transmission electron microscope at 80 kV (JEOL JEM-1400) using a TempCam F416 camera (Tietz Video and Image Processing Systems GmbH, Gauting, Germany). Images were analyzed with Fiji. For contrast purposes, a Gaussian blur was applied.

Microtubule numbers and distances were measured in a single-blind fashion. After all sporozoites with clearly visible microtubules had been defined, the number of microtubules per sporozoite was manually counted. The distance of microtubules to either the IMC or a membrane of a yet-unknown origin were measured by taking the distance from the center of the microtubule to the inner side of the IMC or to the membrane, respectively. A cutoff value of 40 nm was set, with distances of 40 nm or less being considered IMC-close, according to Ferreira et al. (26).

### Fluorescence imaging of P. falciparum-infected erythrocytes.

All fluorescence images were captured using a Zeiss Axioskop 2 Plus microscope with a Hamamatsu digital camera (model C4742-95) or a Leica D6B fluorescence microscope equipped with a Leica DFC9000 GT camera and a Leica Plan Apochromat 100×/1.4 oil objective.

Microscopy of live-parasite-infected erythrocytes was performed as previously described (93). Briefly, parasites were incubated in standard culture medium with 1 μg/mL Hoechst 33342 (Invitrogen) for 15 min at 37°C prior to imaging. A 5.4-μL portion of infected erythrocytes was added on a glass slide and covered with a coverslip. Nuclei were stained with 1 μg/mL Hoechst 33342 (Invitrogen). Microtubules were visualized by incubation of parasites in medium containing 1:1,000 TubulinTracker deep red (Thermo Fisher Scientific; dissolved in dimethyl sulfoxide [DMSO]), which labels polymerized tubulin, for 15 min at 37°C prior to imaging, as previously described (94). Images were processed using Fiji (91), and Adobe Photoshop CC 2021 was used for display purposes only.

### P. falciparum blood stage growth assay.

For growth assays of TGD cell lines, a flow cytometry assay, adapted from previously published assays (95, 96), was performed to measure proliferation over 5 days. Each day, parasite cultures were resuspended, and 20-μL samples were transferred to an Eppendorf tube. Eighty microliters of RPMI containing Hoechst 33342 and dihydroethidium (DHE) was added to obtain final concentrations of 5 μg/mL and 4.5 μg/mL, respectively. Samples were incubated for 20 min (protected from UV light) at room temperature, and parasitemia was determined using an ACEA NovoCyte flow cytometer.

### P. falciparum gametocyte quantification assay.

Giemsa-stained blood smears at day 10 after induction of GDV1 expression were obtained, and at least 10 fields of view were recorded using a 63× objective per treatment and time point. Erythrocyte numbers were then determined using the automated Parasitemia software (http://www.gburri.org/parasitemia/), while the number and morphology of gametocytes was determined manually in 1,096 to 2,632 (mean = 1793) (3D7-iGP-SPM3-GFP-glmS and 3D7-iGP-SPM3-GFP-glmSM9) or 925 to 2,742 (mean = 1,711) (3D7-iGP control and 3D7-iGP-SPM3-TGD) erythrocytes per sample. The counting was performed in a blind fashion.

### P. falciparum GlmS-based gene knockdown.

GlmS-based knockdown assay was adapted from previously published assays (53, 97). To induce knockdown, highly synchronous early ring-stage parasites were split into two dishes, 2.5 mM GLCN was added to one of them, and parasite growth was measured by fluorescence-activated cell sorting (FACS) after two and four parasite replication cycles. Parasite cultures were inspected daily by Giemsa smears and, if necessary, diluted to avoid growth bias caused by high parasitemia. As an additional control, the same amount of glucosamine was also added to 3D7 wild-type parasites. For all analyses, medium was changed daily, and fresh glucosamine was added every day.

GlmS-based knockdown in gametocytes was performed as previously described (98). Briefly, synchronized ring-stage cultures were induced by the addition of Shield-1, as described above. At day 3 postinduction, the culture was spilt into two dishes, and one dish was cultured in the presence of 2.5 mM glucosamine for the remaining 10 days.

### Phylogenetic analysis.

For phylogenetic analysis, we identified sequences of potentially orthologous genes in other species by using a protein-to-protein BLAST search (blastp, default parameters) (99) with the *Pf*SPM3 protein sequence as a query (GenBank accession no. XP_001350024.1). The blastp search was performed on the nonredundant (nr) database but excluding “Plasmodium falciparum” as a species. Selected representative sequences (PRCDC_1326300, PGABG01_1325400, PVX_116515, AK88_00350, PcyM_1231000, PKNH_1201300, PCOAH_00043100, PCHAS_1347100, C922_01771, PVVCY_1304510, PBANKA_1342500, PY17X_1347200, PocGH01_12035100, PmUG01_12037200, PRELSG_1200700, PGAL8A_00239500, and HEP_00010500) were aligned with the NCBI tool Cobalt multiple alignment, and a phylogenetic gene tree (fast minimum evolution method based on Grishin protein sequence distances) was built via the NCBI web tools. Mega X (100) was used for visualization of the gene tree.

### Software.

Statistical analyses were performed with GraphPad Prism version 6 or 8 (GraphPad Software, USA) or RStudio Team (RStudio: Integrated Development Environment for R [2022]; RStudio, PBC, Boston, MA [http://www.rstudio.com/]). Plasmids and oligonucleotides were designed using ApE (101) or SnapGene software version 3.2.1 (Insightful Science [snapgene.com]).

### Animal work and ethics statements.

For *in vivo* experiments, 6- to 8-week-old female Swiss mice obtained from Janvier labs were used. These experiments included parasite propagation and mosquito infections. For transmission experiments used to determine parasite infectivity, 4- to 6-week-old female C57/BL6 mice from Charles River Laboratories were used. All animal experiments were performed under the authorization number G111/20 according to the FELASA-B guidelines and were approved by the appropriate authority, the Regierungspräsidium in Karlsruhe, Germany.

## References

[1] WHO. 2020. World malaria report 2020.

[2] Ollomo B, Durand P, Prugnolle F, Douzery E, Arnathau C, Nkoghe D, Leroy E, Renaud F. 2009. A new malaria agent in African hominids. PLoS Pathog 5:e1000446. doi:10.1371/journal.ppat.1000446. 19478877 PMC2680981

[3] Liu W, Sundararaman SA, Loy DE, Learn GH, Li Y, Plenderleith LJ, Ndjango JBN, Speede S, Atencia R, Cox D, Shaw GM, Ayouba A, Peeters M, Rayner JC, Hahn BH, Sharp PM. 2016. Multigenomic delineation of Plasmodium species of the Laverania subgenus infecting wild-living chimpanzees and gorillas. Genome Biol Evol 8:1929–1939. doi:10.1093/gbe/evw128. 27289102 PMC4943199

[4] Liu W, Sherrill-Mix S, Learn GH, Scully EJ, Li Y, Avitto AN, Loy DE, Lauder AP, Sundararaman SA, Plenderleith LJ, Ndjango JBN, Georgiev AV, Ahuka-Mundeke S, Peeters M, Bertolani P, Dupain J, Garai C, Hart JA, Hart TB, Shaw GM, Sharp PM, Hahn BH. 2017. Wild bonobos host geographically restricted malaria parasites including a putative new Laverania species. Nat Commun 8:1635. doi:10.1038/s41467-017-01798-5. 29158512 PMC5696340

[5] Otto TD, Gilabert A, Crellen T, Böhme U, Arnathau C, Sanders M, Oyola SO, Okouga AP, Boundenga L, Willaume E, Ngoubangoye B, Moukodoum ND, Paupy C, Durand P, Rougeron V, Ollomo B, Renaud F, Newbold C, Berriman M, Prugnolle F. 2018. Genomes of all known members of a Plasmodium subgenus reveal paths to virulent human malaria. Nat Microbiol 3:687–697. doi:10.1038/s41564-018-0162-2. 29784978 PMC5985962

[6] Su XZ, Zhang C, Joy DA. 2020. Host-malaria parasite interactions and impacts on mutual evolution. Front Cell Infect Microbiol 10:587933. doi:10.3389/fcimb.2020.587933. 33194831 PMC7652737

[7] Morrissette NS, Sibley LD. 2002. Cytoskeleton of apicomplexan parasites. Microbiol Mol Biol Rev 66:21–38. doi:10.1128/MMBR.66.1.21-38.2002. 11875126 PMC120781

[8] Garnham PCC, Bird RG, Baker JR, Bray RS. 1961. Electron microscope studies of motile stages of malaria parasites II. The fine structure of the sporozoite of Laverania (= Plasmodium) falcipara. Trans R Soc Trop Med Hyg 55:98–102. doi:10.1016/0035-9203(61)90046-3. 13703708

[9] Garnham PCC, Bird RG, Baker JR. 1962. Electron microscope studies of motile stages of malaria parasites III. The ookinetes of Haemamoeba and Plasmodium. Trans R Soc Trop Med Hyg 56:116–120. doi:10.1016/0035-9203(62)90137-2. 13897014

[10] Garnham PCC, Bird RG, Baker JR. 1963. Electron microscope studies of motile stages of malaria parasites. IV. The fine structure of the sporozoites of four species of plasmodium. Trans R Soc Trop Med Hyg 57:27–31. doi:10.1016/0035-9203(63)90007-5. 13960455

[11] Harding CR, Frischknecht F. 2020. The riveting cellular structures of apicomplexan parasites. Trends Parasitol 36:979–991. doi:10.1016/j.pt.2020.09.001. 33011071

[12] Ferreira JL, Heincke D, Wichers JS, Liffner B, Wilson DW, Gilberger TW. 2020. The dynamic roles of the inner membrane complex in the multiple stages of the malaria parasite. Front Cell Infect Microbiol 10:611801. doi:10.3389/fcimb.2020.611801. 33489940 PMC7820811

[13] Liffner B, Absalon S. 2021. Expansion microscopy reveals Plasmodium falciparum blood-stage parasites undergo anaphase with a chromatin bridge in the absence of mini-chromosome maintenance complex binding protein. Microorganisms 9:2306. doi:10.3390/microorganisms9112306. 34835432 PMC8620465

[14] Bannister LH, Hopkins JM, Fowler RE, Krishna S, Mitchell GH. 2000. A brief illustrated guide to the ultrastructure of Plasmodium falciparum asexual blood stages. Parasitol Today 16:427–433. doi:10.1016/s0169-4758(00)01755-5. 11006474

[15] Kappes B, Rohrbach P. 2007. Microtubule inhibitors as a potential treatment for malaria. Future Microbiol 2:409–423. doi:10.2217/17460913.2.4.409. 17683277

[16] Bertiaux E, Balestra AC, Bournonville L, Louvel V, Maco B, Soldati-Favre D, Brochet M, Guichard P, Hamel V. 2021. Expansion microscopy provides new insights into the cytoskeleton of malaria parasites including the conservation of a conoid. PLoS Biol 19:e3001020. doi:10.1371/journal.pbio.3001020. 33705377 PMC7951857

[17] Li J, Shami GJ, Cho E, Liu B, Hanssen E, Dixon MWA, Tilley L. 2022. Repurposing the mitotic machinery to drive cellular elongation and chromatin reorganisation in Plasmodium falciparum gametocytes. Nat Commun 13:5054. doi:10.1038/s41467-022-32579-4. 36030238 PMC9419145

[18] Simon CS, Funaya C, Bauer J, Voβ Y, Machado M, Penning A, Klaschka D, Cyrklaff M, Kim J, Ganter M, Guizetti J. 2021. An extended DNA-free intranuclear compartment organizes centrosome microtubules in malaria parasites. Life Sci Alliance 4:e202101199. doi:10.26508/lsa.202101199. 34535568 PMC8473725

[19] Sinden RE. 1982. Gametocytogenesis of Plasmodium falciparum in vitro: an electron microscopic study. Parasitology 84:1–11. doi:10.1017/s003118200005160x. 7038594

[20] Parkyn Schneider M, Liu B, Glock P, Suttie A, McHugh E, Andrew D, Batinovic S, Williamson N, Hanssen E, McMillan P, Hliscs M, Tilley L, Dixon MWA. 2017. Disrupting assembly of the inner membrane complex blocks Plasmodium falciparum sexual stage development. PLoS Pathog 13:e1006659. doi:10.1371/journal.ppat.1006659. 28985225 PMC5646874

[21] Rashpa R, Brochet M. 2022. Expansion microscopy of Plasmodium gametocytes reveals the molecular architecture of a bipartite microtubule organisation centre coordinating mitosis with axoneme assembly. PLoS Pathog 18:e1010223. doi:10.1371/journal.ppat.1010223. 35077503 PMC8789139

[22] Dixon MWA, Tilley L. 2021. Plasmodium falciparum goes bananas for sex. Mol Biochem Parasitol 244:111385. doi:10.1016/j.molbiopara.2021.111385. 34062177

[23] Bray RS. 1958. Studies on malaria in chimpanzees. Am J Trop Med Hyg 7:20–24. doi:10.4269/ajtmh.1958.7.20. 13508992

[24] Dixon MWA, Dearnley MK, Hanssen E, Gilberger T, Tilley L. 2012. Shape-shifting gametocytes: how and why does P falciparum go banana-shaped? Trends Parasitol doi:10.1016/j.pt.2012.07.007. 22939181

[25] Ngotho P, Soares AB, Hentzschel F, Achcar F, Bertuccini L, Marti M. 2019. Revisiting gametocyte biology in malaria parasites. FEMS Microbiol Rev 43:401–414. doi:10.1093/femsre/fuz010. 31220244 PMC6606849

[26] Ferreira JL, Pražák V, Vasishtan D, Siggel M, Hentzschel F, Pietsch E, Kosinski J, Frischknecht F, Gilberger TW, Grünewald K. 2022. Form follows function: variable microtubule architecture in the malaria parasite. bioRxiv. doi:10.1101/2022.04.13.488170. PMC9984467 36869034

[27] Tiburcio M, Niang M, Deplaine G, Perrot S, Bischoff E, Ndour PA, Silvestrini F, Khattab A, Milon G, David PH, Hardeman M, Vernick KD, Sauerwein RW, Preiser PR, Mercereau-Puijalon O, Buffet P, Alano P, Lavazec C, Tibúrcio M, Niang M, Deplaine G, Perrot S, Bischoff E, Ndour PA, Silvestrini F, Khattab A, Milon G, David PH, Hardeman M, Vernick KD, Sauerwein RW, Preiser PR, Mercereau-Puijalon O, Buffet P, Alano P, Lavazec C. 2012. A switch in infected erythrocyte deformability at the maturation and blood circulation of Plasmodium falciparum transmission stages. Blood 119:e172–e180. doi:10.1182/blood-2012-03-414557. 22517905 PMC3382942

[28] Aingaran M, Zhang R, Law SK, Peng Z, Undisz A, Meyer E, Diez-Silva M, Burke TA, Spielmann T, Lim CT, Suresh S, Dao M, Marti M. 2012. Host cell deformability is linked to transmission in the human malaria parasite Plasmodium falciparum. Cell Microbiol 14:983–993. doi:10.1111/j.1462-5822.2012.01786.x. 22417683 PMC3376226

[29] De Niz M, Meibalan E, Mejia P, Ma S, Brancucci NMB, Agop-Nersesian C, Mandt R, Ngotho P, Hughes KR, Waters AP, Huttenhower C, Mitchell JR, Martinelli R, Frischknecht F, Seydel KB, Taylor T, Milner D, Heussler VT, Marti M. 2018. Plasmodium gametocytes display homing and vascular transmigration in the host bone marrow. Sci Adv 4:eaat3775. doi:10.1126/sciadv.aat3775. 29806032 PMC5966192

[30] Tran JQ, Li C, Chyan A, Chung L, Morrissette NS. 2012. SPM1 stabilizes subpellicular microtubules in toxoplasma gondii. Eukaryot Cell 11:206–216. doi:10.1128/EC.05161-11. 22021240 PMC3272897

[31] Liu J, Wetzel L, Zhang Y, Nagayasu E, Ems-Mcclung S, Florens L, Hu K. 2013. Novel thioredoxin-like proteins are components of a protein complex coating the cortical microtubules of Toxoplasma gondii. Eukaryot Cell 12:1588–1599. doi:10.1128/EC.00082-13. 23873863 PMC3889574

[32] Liu J, He Y, Benmerzouga I, Sullivan WJ, Morrissette NS, Murray JM, Hu K. 2016. An ensemble of specifically targeted proteins stabilizes cortical microtubules in the human parasite toxoplasma gondii. Mol Biol Cell 27:549–571. doi:10.1091/mbc.E15-11-0754. 26680740 PMC4751604

[33] Morrissette N. 2015. Targeting Toxoplasma tubules: tubulin, microtubules, and associated proteins in a human pathogen. Eukaryot Cell 14:2–12. doi:10.1128/EC.00225-14. 25380753 PMC4279016

[34] Dos Santos Pacheco N, Tosetti N, Koreny L, Waller RF, Soldati-Favre D. 2020. Evolution, composition, assembly, and function of the conoid in apicomplexa. Trends Parasitol 36:688–704. doi:10.1016/j.pt.2020.05.001. 32487504

[35] Sun SY, Segev-Zarko LA, Chen M, Pintilie GD, Schmid MF, Ludtke SJ, Boothroyd JC, Chiu W. 2022. Cryo-ET of Toxoplasma parasites gives subnanometer insight into tubulin-based structures. Proc Natl Acad Sci USA 119:e2111661119. doi:10.1073/pnas.2111661119. 35121661 PMC8832990

[36] Wang X, Fu Y, Beatty WL, Ma M, Brown A, David Sibley L, Zhang R. 2021. Cryo-EM structure of cortical microtubules from human parasite Toxoplasma gondii identifies their microtubule inner proteins. Nat Commun 12:3065. doi:10.1038/s41467-021-23351-1. 34031406 PMC8144581

[37] Tosetti N, Pacheco N dos S, Bertiaux E, Maco B, Bournonville L, Hamel V, Guichard P, Soldati-Favre D. 2020. Essential function of the alveolin network in the subpellicular microtubules and conoid assembly in Toxoplasma gondii. Elife 9:e56635. doi:10.7554/eLife.56635. 32379047 PMC7228768

[38] Leung JM, Nagayasu E, Hwang YC, Liu J, Pierce PG, Phan IQ, Prentice RA, Murray JM, Hu K. 2020. A doublecortin-domain protein of Toxoplasma and its orthologues bind to and modify the structure and organization of tubulin polymers. BMC Mol Cell Biol 21:8. doi:10.1186/s12860-020-0249-5. 32111164 PMC7048138

[39] Cyrklaff M, Kudryashev M, Leis A, Leonard K, Baumeister W, Menard R, Meissner M, Frischknecht F. 2007. Cryoelectron tomography reveals periodic material at the inner side of subpellicular microtubules in apicomplexan parasites. J Exp Med 204:1281–1287. doi:10.1084/jem.20062405. 17562819 PMC2118598

[40] Kudryashev M, Münter S, Lemgruber L, Montagna G, Stahlberg H, Matuschewski K, Meissner M, Cyrklaff M, Frischknecht F. 2012. Structural basis for chirality and directional motility of Plasmodium sporozoites. Cell Microbiol 14:1757–1768. doi:10.1111/j.1462-5822.2012.01836.x. 22776715 PMC4116596

[41] Wichers JS, Wunderlich J, Heincke D, Pazicky S, Strauss J, Schmitt M, Kimmel J, Wilcke L, Scharf S, von Thien H, Burda P, Spielmann T, Löw C, Filarsky M, Bachmann A, Gilberger TW. 2021. Identification of novel inner membrane complex and apical annuli proteins of the malaria parasite Plasmodium falciparum. Cell Microbiol 23:e13341. doi:10.1111/cmi.13341. 33830607

[42] Birnbaum J, Flemming S, Reichard N, Soares AB, Mesén-Ramírez P, Jonscher E, Bergmann B, Spielmann T. 2017. A genetic system to study Plasmodium falciparum protein function. Nat Methods 14:450–456. doi:10.1038/nmeth.4223. 28288121

[43] Wichers JS, Scholz JAM, Strauss J, Witt S, Lill A, Ehnold L-I, Neupert N, Liffner B, Lühken R, Petter M, Lorenzen S, Wilson DW, Löw C, Lavazec C, Bruchhaus I, Tannich E, Gilberger TW, Bachmann A. 2019. Dissecting the gene expression, localization, membrane topology, and function of the Plasmodium falciparum STEVOR protein family. mBio 10:e01500-19. doi:10.1128/mBio.01500-19. 31363031 PMC6667621

[44] Subudhi AK, O’Donnell AJ, Ramaprasad A, Abkallo HM, Kaushik A, Ansari HR, Abdel-Haleem AM, Ben Rached F, Kaneko O, Culleton R, Reece SE, Pain A. 2020. Malaria parasites regulate intra-erythrocytic development duration via serpentine receptor 10 to coordinate with host rhythms. Nat Commun 11:2763. doi:10.1038/s41467-020-16593-y. 32488076 PMC7265539

[45] Hanssen E, Dekiwadia C, Riglar DT, Rug M, Lemgruber L, Cowman AF, Cyrklaff M, Kudryashev M, Frischknecht F, Baum J, Ralph SA. 2013. Electron tomography of Plasmodium falciparum merozoites reveals core cellular events that underpin erythrocyte invasion. Cell Microbiol 15:1457–1472. doi:10.1111/cmi.12132. 23461734

[46] Cabrera A, Herrmann S, Warszta D, Santos JM, John Peter AT, Kono M, Debrouver S, Jacobs T, Spielmann T, Ungermann C, Soldati-Favre D, Gilberger TW. 2012. Dissection of minimal sequence requirements for rhoptry membrane targeting in the malaria parasite. Traffic 13:1335–1350. doi:10.1111/j.1600-0854.2012.01394.x. 22759070

[47] Geiger M, Brown C, Wichers JS, Strauss J, Lill A, Thuenauer R, Liffner B, Wilcke L, Lemcke S, Heincke D, Pazicky S, Bachmann A, Löw C, Wilson DW, Filarsky M, Burda P-C, Zhang K, Junop M, Gilberger TW. 2020. Structural insights into PfARO and characterization of its interaction with PfAIP. J Mol Biol 432:878–896. doi:10.1016/j.jmb.2019.12.024. 31877322

[48] Gould SB, Tham W-HH, Cowman AF, McFadden GI, Waller RF. 2008. Alveolins, a new family of cortical proteins that define the protist infrakingdom Alveolata. Mol Biol Evol 25:1219–1230. doi:10.1093/molbev/msn070. 18359944

[49] Anderson-White BR, Ivey FD, Cheng K, Szatanek T, Lorestani A, Beckers CJ, Ferguson DJP, Sahoo N, Gubbels MJ. 2011. A family of intermediate filament-like proteins is sequentially assembled into the cytoskeleton of Toxoplasma gondii. Cell Microbiol 13:18–31. doi:10.1111/j.1462-5822.2010.01514.x. 20698859 PMC3005026

[50] Saini E, Zeeshan M, Brady D, Pandey R, Kaiser G, Koreny L, Kumar P, Thakur V, Tatiya S, Katris NJ, Limenitakis RS, Kaur I, Green JL, Bottrill AR, Guttery DS, Waller RF, Heussler V, Holder AA, Mohmmed A, Malhotra P, Tewari R. 2017. Photosensitized INA-labelled protein 1 (PhIL1) is novel component of the inner membrane complex and is required for Plasmodium parasite development. Sci Rep 7:15577. doi:10.1038/s41598-017-15781-z. 29138437 PMC5686188

[51] Saini E, Sheokand PK, Sharma V, Agrawal P, Kaur I, Singh S, Mohmmed A, Malhotra P. 2021. Plasmodium falciparum PhIL1-associated complex plays an essential role in merozoite reorientation and invasion of host erythrocytes. PLoS Pathog 17:e1009750. doi:10.1371/journal.ppat.1009750. 34324609 PMC8321122

[52] Kono M, Herrmann S, Loughran NB, Cabrera A, Engelberg K, Lehmann C, Sinha D, Prinz B, Ruch U, Heussler V, Spielmann T, Parkinson J, Gilberger TW. 2012. Evolution and architecture of the inner membrane complex in asexual and sexual stages of the malaria parasite. Mol Biol Evol 29:2113–2132. doi:10.1093/molbev/mss081. 22389454

[53] Prommana P, Uthaipibull C, Wongsombat C, Kamchonwongpaisan S, Yuthavong Y, Knuepfer E, Holder AA, Shaw PJ. 2013. Inducible knockdown of Plasmodium gene expression using the glmS ribozyme. PLoS One 8:e73783. doi:10.1371/journal.pone.0073783. 24023691 PMC3758297

[54] Boltryk SD, Passecker A, Alder A, Carrington E, van de Vegte-Bolmer M, van Gemert G-J, van der Starre A, Beck H-P, Sauerwein RW, Kooij TWA, Brancucci NMB, Proellochs NI, Gilberger T-W, Voss TS. 2021. CRISPR/Cas9-engineered inducible gametocyte producer lines as a valuable tool for Plasmodium falciparum malaria transmission research. Nat Commun 12:4806. doi:10.1038/s41467-021-24954-4. 34376675 PMC8355313

[55] Ripp J, Smyrnakou X, Neuhoff M, Hentzschel F, Frischknecht F. 2022. Phosphorylation of myosin A regulates gliding motility and is essential for Plasmodium transmission. EMBO Rep 23:e54857. doi:10.15252/embr.202254857. 35506479 PMC9253774

[56] Kehrer J, Formaglio P, Muthinja JM, Weber S, Baltissen D, Lance C, Ripp J, Grech J, Meissner M, Funaya C, Amino R, Frischknecht F. 2022. Plasmodium sporozoite disintegration during skin passage limits malaria parasite transmission. EMBO Rep 23:e54719. doi:10.15252/embr.202254719. 35403820 PMC9253755

[57] Aleshnick M, Ganusov VV, Nasir G, Yenokyan G, Sinnis P. 2020. Experimental determination of the force of malaria infection reveals a non-linear relationship to mosquito sporozoite loads. PLoS Pathog 16:e1008181. doi:10.1371/journal.ppat.1008181. 32453765 PMC7295235

[58] Clements RL, Morano AA, Navarro FM, McGee JP, Du EW, Streva VA, Lindner SE, Dvorin JD. 2022. Identification of basal complex protein that is essential for maturation of transmission-stage malaria parasites. Proc Natl Acad Sci USA 119:e2204167119. doi:10.1073/pnas.2204167119. 35972967 PMC9407223

[59] Zhang M, Wang C, Otto TD, Oberstaller J, Liao X, Adapa SR, Udenze K, Bronner IF, Casandra D, Mayho M, Brown J, Li S, Swanson J, Rayner JC, Jiang RHY, Adams JH. 2018. Uncovering the essential genes of the human malaria parasite Plasmodium falciparum by saturation mutagenesis. Science 360:eaap7847. doi:10.1126/science.aap7847. 29724925 PMC6360947

[60] Bushell E, Gomes AR, Sanderson T, Anar B, Girling G, Herd C, Metcalf T, Modrzynska K, Schwach F, Martin RE, Mather MW, McFadden GI, Parts L, Rutledge GG, Vaidya AB, Wengelnik K, Rayner JC, Billker O. 2017. Functional profiling of a Plasmodium genome reveals an abundance of essential genes. Cell 170:260–272.E8. doi:10.1016/j.cell.2017.06.030. 28708996 PMC5509546

[61] Aikawa M, Huff CG, Sprinz H. 1969. Comparative fine structure study of the gametocytes of avian, reptilian, and mammalian malarial parasites. J Ultrastruct Res 26:316–331. doi:10.1016/s0022-5320(69)80010-9. 4887539

[62] Garnham PCC. 1966. Malaria parasites and other haemosporidia. Blackwell Scientific Publications, Oxford, United Kingdom.

[63] Spreng B, Fleckenstein H, Kübler P, Di Biagio C, Benz M, Patra P, Schwarz US, Cyrklaff M, Frischknecht F. 2019. Microtubule number and length determine cellular shape and function in Plasmodium. EMBO J 38:100984. doi:10.15252/embj.2018100984. PMC6669926 31368598

[64] Frixione E, Mondragón R, Meza I. 1996. Kinematic analysis of Toxoplasma gondii motility. Cell Motil Cytoskeleton 34:152–163. doi:10.1002/(SICI)1097-0169(1996)34:2<152::AID-CM6>3.0.CO;2-D. 8769726

[65] Speer CA, Rosales-Ronquillo MC, Silverman PH. 1975. Motility of Plasmodium berghei ookinetes in vitro. J Invertebr Pathol 25:73–78. doi:10.1016/0022-2011(75)90286-4. 1089734

[66] Howick VM, Russell AJC, Andrews T, Heaton H, Reid AJ, Natarajan K, Butungi H, Metcalf T, Verzier LH, Rayner JC, Berriman M, Herren JK, Billker O, Hemberg M, Talman AM, Lawniczak MKN. 2019. The malaria cell atlas: single parasite transcriptomes across the complete Plasmodium life cycle. Science 365:eaaw2619. doi:10.1126/science.aaw2619. 31439762 PMC7056351

[67] Reid AJ, Talman AM, Bennett HM, Gomes AR, Sanders MJ, Illingworth CJR, Billker O, Berriman M, Lawniczak MKN. 2018. Single-cell RNA-seq reveals hidden transcriptional variation in malaria parasites. Elife 7:e33105. doi:10.7554/eLife.33105. 29580379 PMC5871331

[68] Moreau CA, Bhargav SP, Kumar H, Quadt KA, Piirainen H, Strauss L, Kehrer J, Streichfuss M, Spatz JP, Wade RC, Kursula I, Frischknecht F. 2017. A unique profilin-actin interface is important for malaria parasite motility. PLoS Pathog 13:e1006412. doi:10.1371/journal.ppat.1006412. 28552953 PMC5464670

[69] Douglas RG, Nandekar P, Aktories JE, Kumar H, Weber R, Sattler JM, Singer M, Lepper S, Sadiq SK, Wade RC, Frischknecht F. 2018. Inter-subunit interactions drive divergent dynamics in mammalian and Plasmodium actin filaments. PLoS Biol 16:e2005345. doi:10.1371/journal.pbio.2005345. 30011270 PMC6055528

[70] Yee M, Walther T, Frischknecht F, Douglas RG. 2022. Divergent Plasmodium actin residues are essential for filament localization, mosquito salivary gland invasion and malaria transmission. PLoS Pathog 18:e1010779. doi:10.1371/journal.ppat.1010779. 35998188 PMC9439217

[71] Vanderberg J, Rdodin J, Yoeli M. 1967. Electron microscopic and histochemical studies of sporozoite formation in Plasmodium berghei. J Protozool 14:82–103. doi:10.1111/j.1550-7408.1967.tb01452.x.

[72] Moon RW, Taylor CJ, Bex C, Schepers R, Goulding D, Janse CJ, Waters AP, Baker DA, Billker O. 2009. A cyclic GMP signalling module that regulates gliding motility in a malaria parasite. PLoS Pathog 5:e1000599. doi:10.1371/journal.ppat.1000599. 19779564 PMC2742896

[73] Amino R, Thiberge S, Martin B, Celli S, Shorte S, Frischknecht F, Ménard R. 2006. Quantitative imaging of Plasmodium transmission from mosquito to mammal. Nat Med 12:220–224. doi:10.1038/nm1350. 16429144

[74] Hopp CS, Chiou K, Ragheb DRT, Salman AM, Khan SM, Liu AJ, Sinnis P. 2015. Longitudinal analysis of plasmodium sporozoite motility in the dermis reveals component of blood vessel recognition. Elife 4:e07789. doi:10.7554/eLife.07789. 26271010 PMC4594146

[75] Harding CR, Gow M, Kang JH, Shortt E, Manalis SR, Meissner M, Lourido S. 2019. Alveolar proteins stabilize cortical microtubules in Toxoplasma gondii. Nat Commun 10:401. doi:10.1038/s41467-019-08318-7. 30674885 PMC6344517

[76] Walliker D, Quakyi IA, Wellems TE, McCutchan TF, Szarfman A, London WT, Corcoran LM, Burkot TR, Carter R. 1987. Genetic analysis of the human malaria parasite Plasmodium falciparum. Science 236:1661–1666. doi:10.1126/science.3299700. 3299700

[77] Trager W, Jensen JB. 1997. Continuous culture of Plasmodium falciparum: its impact on malaria research. Int J Parasitol 27:989–1006. doi:10.1016/s0020-7519(97)00080-5. 9363481

[78] Rivadeneira E, Wasserman M, Espinal C. 1983. Separation and concentration of schizonts of Plasmodium falciparum by Percoll gradients. J Protozool 30:367–370. doi:10.1111/j.1550-7408.1983.tb02932.x. 6313915

[79] Lambros C, Vanderberg JP. 1979. Synchronization of Plasmodium falciparum erythrocytic stages in culture. J Parasitol 65:418–420. doi:10.2307/3280287. 383936

[80] Filarsky M, Fraschka SA, Niederwieser I, Brancucci NMB, Carrington E, Carrió E, Moes S, Jenoe P, Bártfai R, Voss TS. 2018. GDV1 induces sexual commitment of malaria parasites by antagonizing HP1-dependent gene silencing. Science 359:1259–1263. doi:10.1126/science.aan6042. 29590075 PMC6219702

[81] Ponnudurai T, Lensen AHW, Meis JFGM, Meuwissen JHE. 1986. Synchronization of Plasmodium falciparum gametocytes using an automated suspension culture system. Parasitology 93:263–274. doi:10.1017/S003118200005143X. 3537921

[82] Winkler WC, Nahvi A, Roth A, Collins JA, Breaker RR. 2004. Control of gene expression by a natural metabolite-responsive ribozyme. Nature 428:281–286. doi:10.1038/nature02362. 15029187

[83] Wichers JS, Mesén-Ramírez P, Fuchs G, Yu-Strzelczyk J, Stäcker J, von Thien H, Alder A, Henshall I, Liffner B, Nagel G, Löw C, Wilson D, Spielmann T, Gao S, Gilberger T-W, Bachmann A, Strauss J. 2022. PMRT1, a Plasmodium-specific parasite plasma membrane transporter, is essential for asexual and sexual blood stage development. mBio 13:e00623-22. doi:10.1128/mbio.00623-22. 35404116 PMC9040750

[84] Beyer K, Kracht S, Kehrer J, Singer M, Klug D, Frischknecht F. 2021. Limited Plasmodium sporozoite gliding motility in the absence of TRAP family adhesins. Malar J 20:430. doi:10.1186/s12936-021-03960-3. 34717635 PMC8557484

[85] Kehrer J, Ricken D, Strauss L, Pietsch E, Heinze JM, Frischknecht F. 2020. APEX-based proximity labeling in Plasmodium identifies a membrane protein with dual functions during mosquito infection. bioRxiv. https://www.biorxiv.org/content/10.1101/2020.09.29.318857v1. 10.1371/journal.ppat.1012788 PMC11695019 39693377

[86] Moon RW, Hall J, Rangkuti F, Ho YS, Almond N, Mitchell GH, Pain A, Holder AA, Blackman MJ. 2013. Adaptation of the genetically tractable malaria pathogen Plasmodium knowlesi to continuous culture in human erythrocytes. Proc Natl Acad Sci USA 110:531–536. doi:10.1073/pnas.1216457110. 23267069 PMC3545754

[87] Ganesan SM, Morrisey JM, Ke H, Painter HJ, Laroiya K, Phillips MA, Rathod PK, Mather MW, Vaidya AB. 2011. Yeast dihydroorotate dehydrogenase as a new selectable marker for Plasmodium falciparum transfection. Mol Biochem Parasitol 177:29–34. doi:10.1016/j.molbiopara.2011.01.004. 21251930 PMC3057331

[88] Janse CJ, Ramesar J, Waters AP. 2006. High-efficiency transfection and drug selection of genetically transformed blood stages of the rodent malaria parasite Plasmodium berghei. Nat Protoc 1:346–356. doi:10.1038/nprot.2006.53. 17406255

[89] Spaccapelo R, Janse CJ, Caterbi S, Franke-Fayard B, Bonilla JA, Syphard LM, Di Cristina M, Dottorini T, Savarino A, Cassone A, Bistoni F, Waters AP, Dame JB, Crisanti A. 2010. Plasmepsin 4-deficient Plasmodium berghei are virulence attenuated and induce protective immunity against experimental malaria. Am J Pathol 176:205–217. doi:10.2353/ajpath.2010.090504. 20019192 PMC2797883

[90] Klug D, Mair GR, Frischknecht F, Douglas RG. 2016. A small mitochondrial protein present in myzozoans is essential for malaria transmission. Open Biol 6:160034. doi:10.1098/rsob.160034. 27053680 PMC4852462

[91] Schindelin J, Arganda-Carreras I, Frise E, Kaynig V, Longair M, Pietzsch T, Preibisch S, Rueden C, Saalfeld S, Schmid B, Tinevez J-Y, White DJ, Hartenstein V, Eliceiri K, Tomancak P, Cardona A. 2012. Fiji: an open-source platform for biological-image analysis. Nat Methods 9:676–682. doi:10.1038/nmeth.2019. 22743772 PMC3855844

[92] Hegge S, Kudryashev M, Smith A, Frischknecht F. 2009. Automated classification of Plasmodium sporozoite movement patterns reveals a shift towards productive motility during salivary gland infection. Biotechnol J 4:903–913. doi:10.1002/biot.200900007. 19455538

[93] Grüring C, Spielmann T. 2012. Imaging of live malaria blood stage parasites. Methods Enzymol 506:81–92. doi:10.1016/B978-0-12-391856-7.00029-9. 22341220

[94] Kimmel J, Schmitt M, Sinner A, Jansen P, Mainye S, Ramón-Zamorano G, Toenhake CG, Wichers JS, Cronshagen J, Sabitzki R, Mesén-Ramírez P, Behrens HM, Bártfai R, Spielmann T. 2022. Gene-by-gene screen of the unknown proteins encoded on P falciparum chromosome 3. bioRxiv. https://www.biorxiv.org/content/10.1101/2022.07.07.499005v1. 10.1016/j.cels.2022.12.001 36657393

[95] Malleret B, Claser C, Ong ASM, Suwanarusk R, Sriprawat K, Howland SW, Russell B, Nosten F, Rénia L. 2011. A rapid and robust tri-color flow cytometry assay for monitoring malaria parasite development. Sci Rep 1:118. doi:10.1038/srep00118. 22355635 PMC3216599

[96] Jonscher E, Flemming S, Schmitt M, Sabitzki R, Reichard N, Birnbaum J, Bergmann B, Höhn K, Spielmann T. 2019. PfVPS45 is required for host cell cytosol uptake by malaria blood stage parasites. Cell Host Microbe 25:166–173.E5. doi:10.1016/j.chom.2018.11.010. 30581113

[97] Burda P-C, Crosskey T, Lauk K, Zurborg A, Söhnchen C, Liffner B, Wilcke L, Strauss J, Jeffries CM, Svergun DI, Wilson DW, Wilmanns M, Gilberger T-W, Pietsch E, Strauss J, Jeffries CM, Svergun DI, Wilson DW, Wilmanns M, Gilberger T-W. 2020. Structure-based identification and functional characterization of a lipocalin in the malaria parasite Plasmodium falciparum. Cell Rep 31:107817. doi:10.1016/j.celrep.2020.107817. 32579913

[98] Wichers JS, van Gelder C, Fuchs G, Ruge JM, Pietsch E, Ferreira JL, Safavi S, von Thien H, Burda P-C, Mesén-Ramirez P, Spielmann T, Strauss J, Gilberger T-W, Bachmann A. 2021. Characterization of Apicomplexan amino acid transporters (ApiATs) in the malaria parasite Plasmodium falciparum. mSphere 6:e00743-21. doi:10.1128/mSphere.00743-21. 34756057 PMC8579892

[99] Altschul SF, Madden TL, Schäffer AA, Zhang J, Zhang Z, Miller W, Lipman DJ. 1997. Gapped BLAST and PSI-BLAST: a new generation of protein database search programs. Nucleic Acids Res 25:3389–3402. doi:10.1093/nar/25.17.3389. 9254694 PMC146917

[100] Kumar S, Stecher G, Li M, Knyaz C, Tamura K. 2018. MEGA X: molecular evolutionary genetics analysis across computing platforms. Mol Biol Evol 35:1547–1549. doi:10.1093/molbev/msy096. 29722887 PMC5967553

[101] Davis MW, Jorgensen EM. 2022. ApE, A plasmid Editor: a freely available DNA manipulation and visualization program. Front Bioinforma 2:818619. doi:10.3389/fbinf.2022.818619. PMC9580900 36304290

