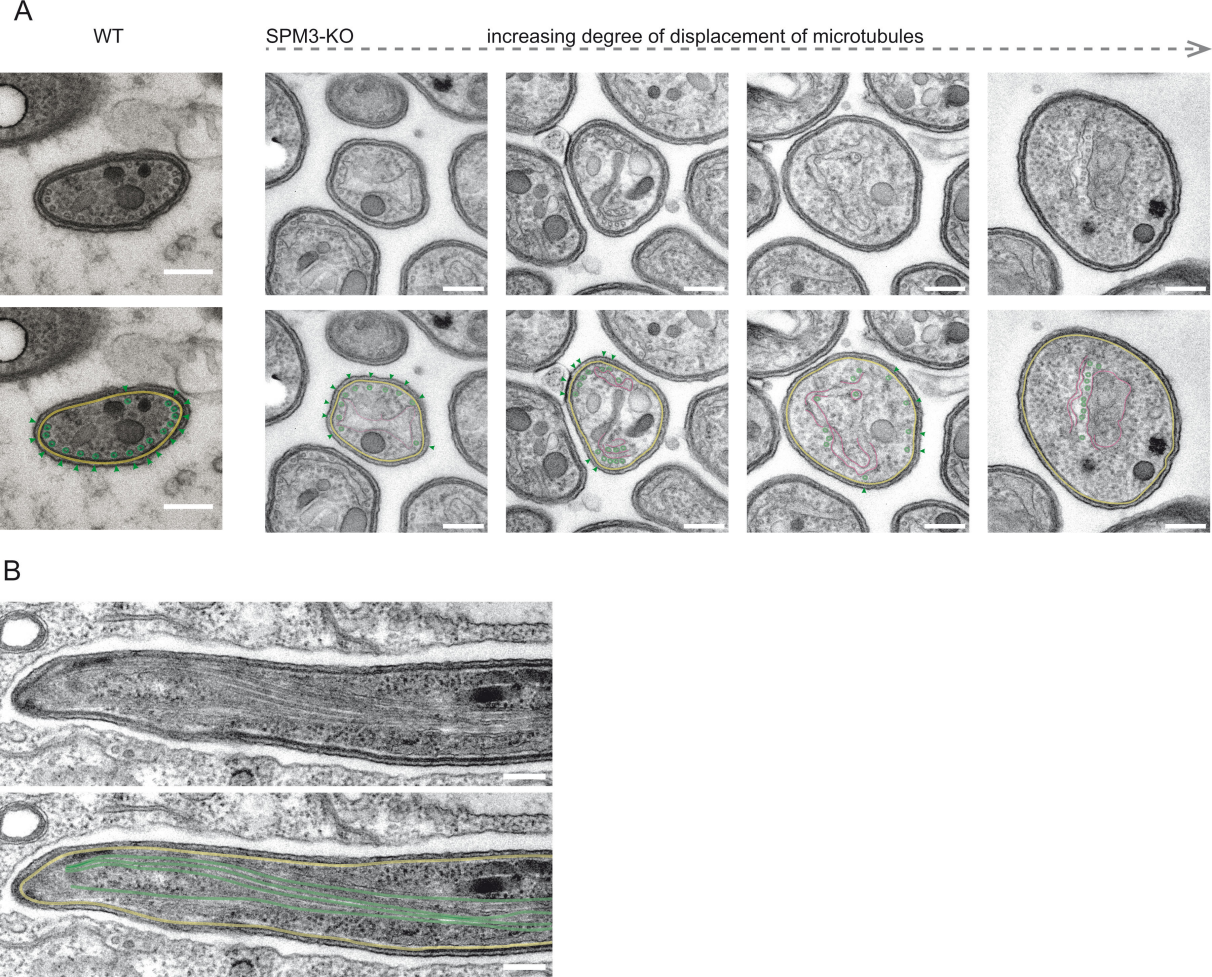# Erratum for Wichers-Misterek et al., “A Microtubule-Associated Protein Is Essential for Malaria Parasite Transmission”

**DOI:** 10.1128/mbio.01786-23

**Published:** 2023-09-29

**Authors:** Jan Stephan Wichers-Misterek, Annika M. Binder, Paolo Mesén-Ramírez, Lilian Patrick Dorner, Soraya Safavi, Gwendolin Fuchs, Tobias L. Lenz, Anna Bachmann, Danny Wilson, Friedrich Frischknecht, Tim-Wolf Gilberger

## ERRATUM

Volume 14, no. 1, e03318-23, 2023, https://doi.org/10.1128/mbio.03318-22. Page 10, Fig. 5A and B: Parts of the colored markups (yellow, green, and magenta) are missing. The panels should appear as shown below.

**Fig 5:**