# Supplementary figures and images for: A Microtubule-Associated Protein Is Essential for Malaria Parasite Transmission
Source: mBio. 2023 Jan 10;14(1):e03318-22. doi: 10.1128/mbio.03318-22 (PMC9973338; doi:10.1128/mbio.03318-22)

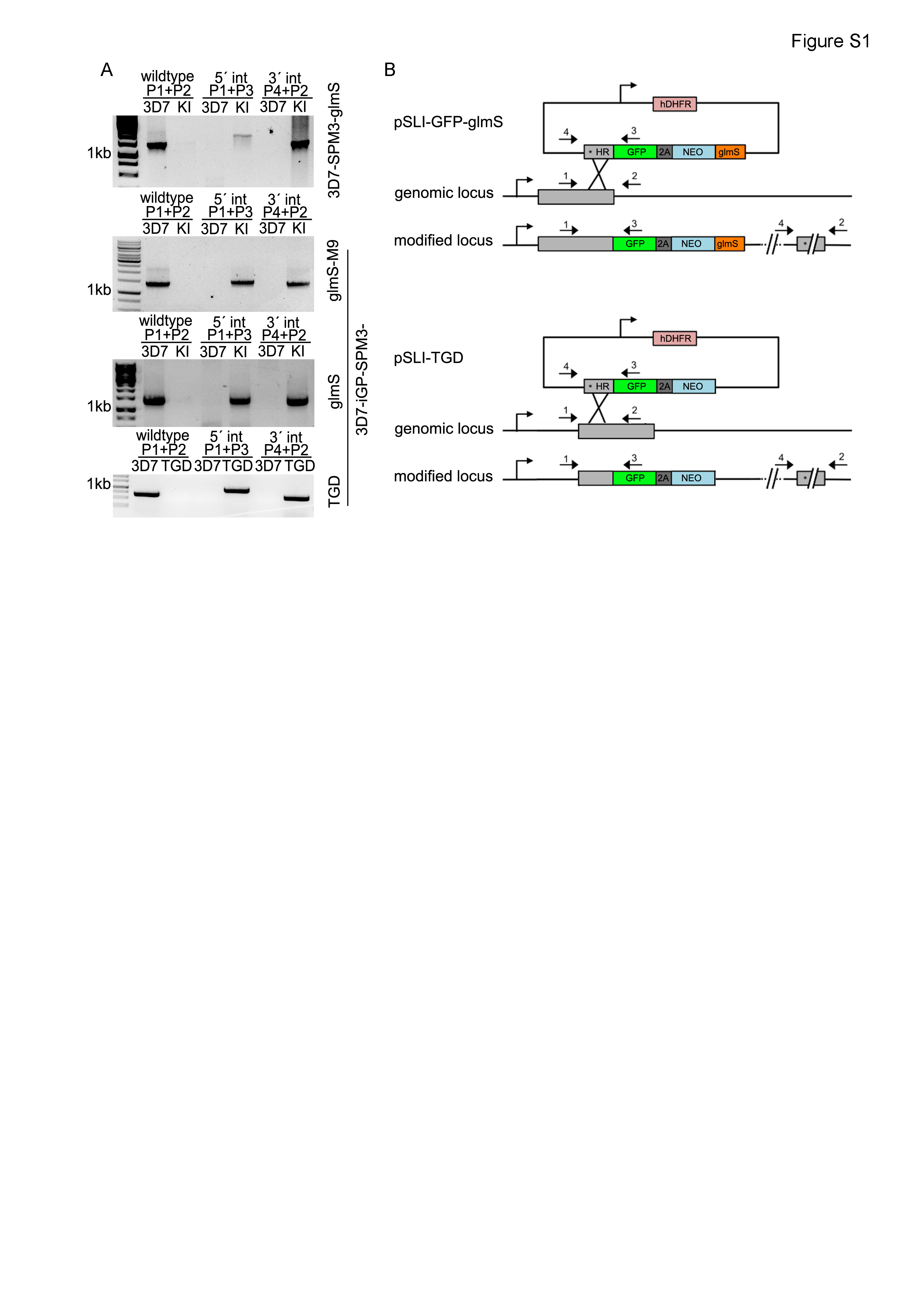

Supplement: FIG S1 [file mbio.03318-22-s0001.tif]

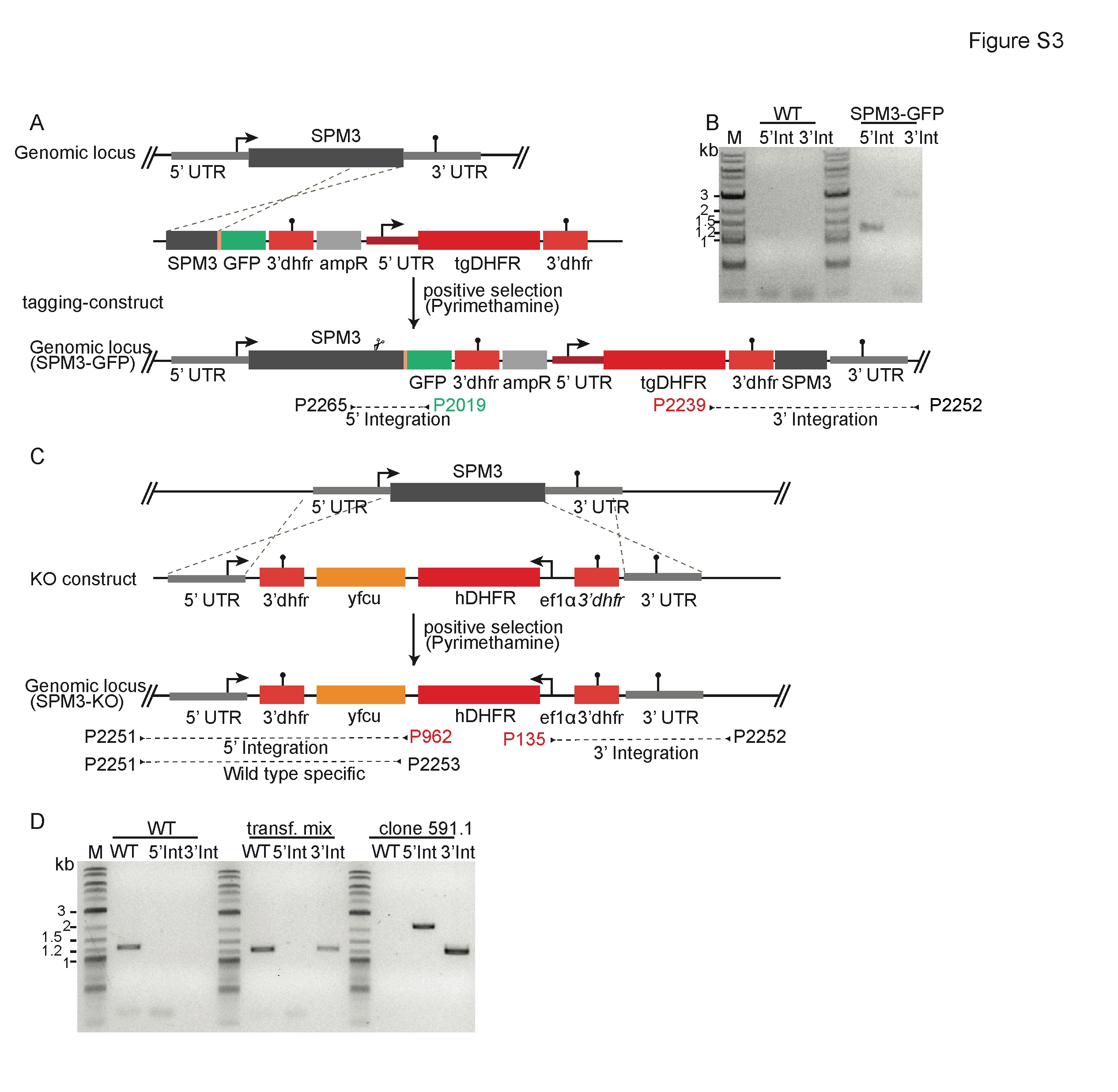

Supplement: FIG S3 [file mbio.03318-22-s0003.tif]

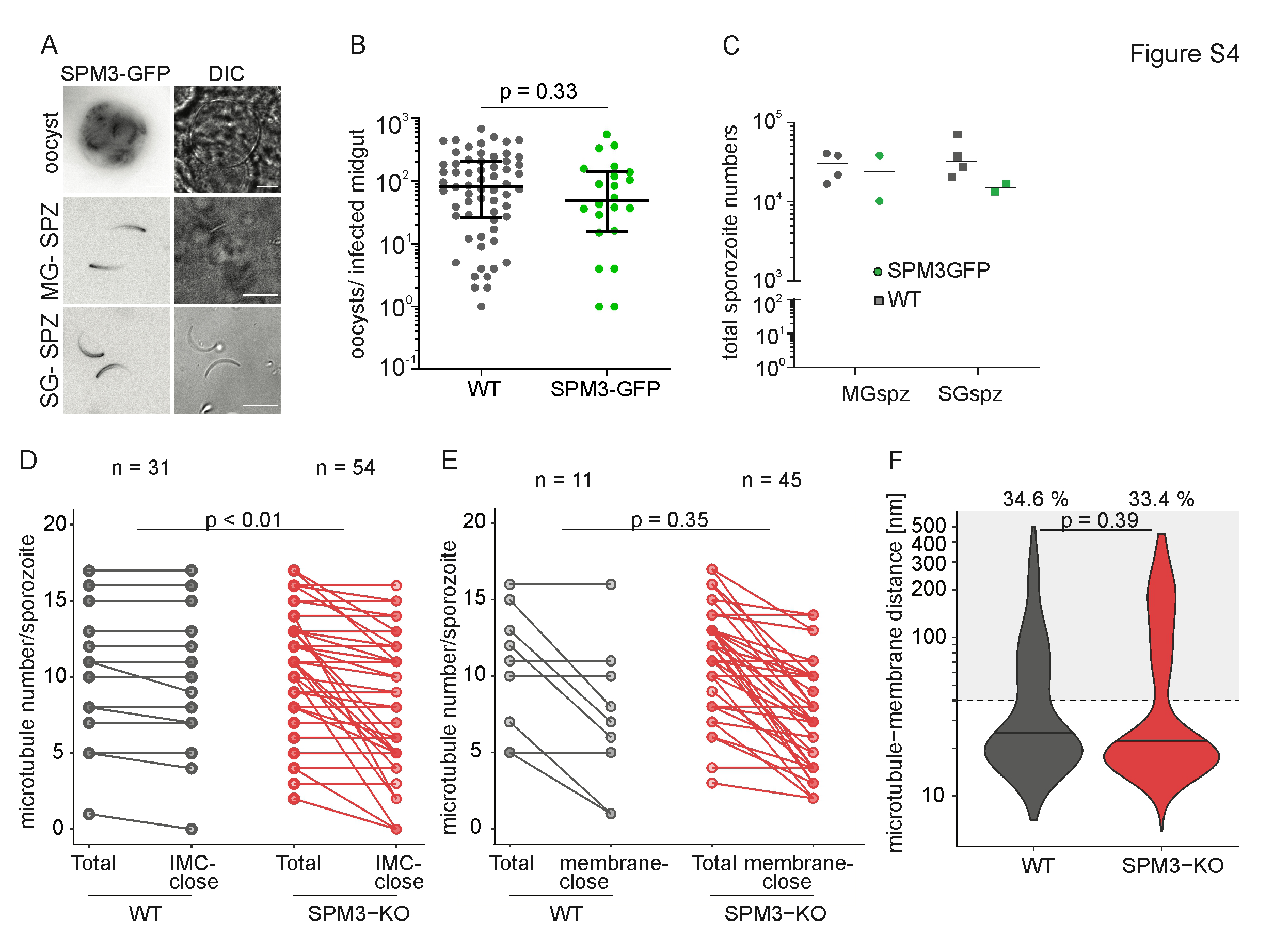

Supplement: FIG S4 [file mbio.03318-22-s0004.tif]
