# Supplementary material for: A Microtubule-Associated Protein Is Essential for Malaria Parasite Transmission
Source: mBio. 2023 Jan 10;14(1):e03318-22. doi: 10.1128/mbio.03318-22 (PMC9973338; doi:10.1128/mbio.03318-22)
Supplement: TABLE S1 [file mbio.03318-22-s0009.pdf]

Table S1: Oligonucleotides used

ID	Target or Primer name	Sequence	Purpose
226	GFP rv	TTTTGTTGATAATGGTCTGC	integration check PCRs
272as	GFP as 272	CCTTCGGGCATGGCACTC	
238	pARL sense 55	GGAATTGTGAGCGGATAACAATTTCACACAGG	
539	Pf3d7_1327300 int fw	GGTAACAGGGAATATAAGCAAG	
540	Pf3d7_1327300 int rv	TATAGGTATACATATTAAGC	
JSW 47	TGD intcheck fw	CCATACATATTTCACACAAATTTAT	
JSW 48	TGD intcheck rv	CATTTAAAGAAAAGCTTCCCCAGG	
547	Pf3d7_1327300_TGD fw	GGGgcggccgctaaGATAATATAAATGCGGATCTATC	cloning of *P.falciparum* constructs
548	Pf3d7_1327300_TGD rv	GGGacgcgtATCAATAATAACATGAGTATTTTC	
533	Pf3d7_1327300 fw	GGGgcggccgctaaCGATGGTTATGAAGGTATTGCTAAAAG	
534	Pf3d7_1327300 rv	GGGacgcgtAATGCAATTACAATAATTCGTTGTTG	
478	PhIL1 fw	GGGggtaccATGCTTTCTTCCATATCACC	
479	PhIL1 rv	GGGcctaggCATATCTTGGTTATAATTTTCTTG	
JSW 2	spm1 FL KPNI	GGGggtaccATGGAAGTTATAACAGAAAAAC	
JSW 3	spm1 FL AVRII	GGGcctaggGTACCACTTTTTTGTTTCTC	
JSW 18	PF3D7_1345600 fw	GGGggtaccATGGAGAAAATAGAGAAAAAATTTTGATG	
JSW 13	PF3D7_1345600 rv	GGGcctaggTATCAATACTGTATTATCCTTAAC	
P135	PeFIa_5UTRrev	GTAAACTTAAGCATAAAGAGCTCG	Genotyping primers
P962	yFCU_seq_R_2	GAAGAATGATGCTAGCGGAGG	
P2019	GFP_rev	ACCATCTAATTCAACAAGAATTGG	
P2239	pL18-tgdhfr-seq-AB	ACTTTAGAGGCCATGAAGAG	
P2251	PBANKA_1342500_GT-AB	CCACCCAATAATATTTTTCGTATATACG	
P2252	PBANKA_1342500_GT2-AB	TTCACGGGATAATAAAAATTGCTC	
P2253	PBANKA_1342500_QCR1-AB	CACTTCAATGTATAATGGGCC	
P2265	PBANKA_1342500_SPM3-QCR	CAGGAGGAGCTAATGCAATGC	
AB-31	SPM3-fw-EcoRI	CCGAGAATTCCTAATAATAATTCCCATTCTGGTG	cloning of *P.berghei* constructs
AB-32	SPM3-rev-L1-BamHI	CGCGGATCCcgtacgtctacctgcacctccagcaccagcagcagcacctctagcacgcgtcctaggAATACAACTACAATAATTCATTGTT	
P2246	SPM3-5UTR-fw_EcoRI-AB	CCGGAATTCGTATAACATGTGTAGCTAACGG	
P2247	SPM3-5UTR-rev_EcoRV-AB	AAAGATATCTACTAGTGTGTTTATTCACTACAAC	
P2248	SPM3-3UTR-fw_HindIII-AB	CCCAAGCTTACGGGAAAGTGAAAAACG	
P2249	SPM3-3UTR-rev_XhoI-AB	CCGCTCGAGGGTTTTAAGTTAAACATACGAGG	
